# Evolution of sex-specific pace-of-life syndromes: genetic architecture and physiological mechanisms

**DOI:** 10.1007/s00265-018-2462-1

**Published:** 2018-03-16

**Authors:** Elina Immonen, Anni Hämäläinen, Wiebke Schuett, Maja Tarka

**Affiliations:** 1 Department of Ecology and Genetics, Evolutionary Biology Centre (EBC), Uppsala University, Norbyvägen 18 D, SE-75 236 Uppsala, Sweden; 2 Department of Biological Sciences, University of Alberta, Edmonton, T6G 2E9 Canada; 3 Zoological Institute, University of Hamburg, Martin-Luther-King Platz 3, 20146 Hamburg, Germany; 4 Center for Biodiversity Dynamics, Department of Biology, Norwegian University of Science and Technology (NTNU), Høgskoleringen 5, 7491 Trondheim, Norway

**Keywords:** Sexual conflict, Sexual dimorphism, Genetic architecture, Personality, Life history evolution, Physiology

## Abstract

Sex differences in life history, physiology, and behavior are nearly ubiquitous across taxa, owing to sex-specific selection that arises from different reproductive strategies of the sexes. The pace-of-life syndrome (POLS) hypothesis predicts that most variation in such traits among individuals, populations, and species falls along a slow-fast pace-of-life continuum. As a result of their different reproductive roles and environment, the sexes also commonly differ in pace-of-life, with important consequences for the evolution of POLS. Here, we outline mechanisms for how males and females can evolve differences in POLS traits and in how such traits can covary differently despite constraints resulting from a shared genome. We review the current knowledge of the genetic basis of POLS traits and suggest candidate genes and pathways for future studies. Pleiotropic effects may govern many of the genetic correlations, but little is still known about the mechanisms involved in trade-offs between current and future reproduction and their integration with behavioral variation. We highlight the importance of metabolic and hormonal pathways in mediating sex differences in POLS traits; however, there is still a shortage of studies that test for sex specificity in molecular effects and their evolutionary causes. Considering whether and how sexual dimorphism evolves in POLS traits provides a more holistic framework to understand how behavioral variation is integrated with life histories and physiology, and we call for studies that focus on examining the sex-specific genetic architecture of this integration.

**Electronic supplementary material:**

The online version of this article (10.1007/s00265-018-2462-1) contains supplementary material, which is available to authorized users.

## Introduction

Sex differences in life history, behavior, and physiology are nearly ubiquitous in nature (e.g., Lee 2006; Fairbairn et al. 2007; Restif and Amos 2010; Maklakov and Lummaa 2013; Adler and Bonduriansky 2014). Building on life history theory (Stearns 1992), the pace-of-life syndrome (POLS) framework predicts that life history and consistent behavioral differences (“personalities,” sensu Dall et al. 2004, that differ among individuals creating a behavioral syndrome, sensu Sih et al. 2004) covary together with physiology along a fast-slow continuum across individuals, populations, and species as a result of trade-offs between reproduction and survival or future reproductive rate (Ricklefs and Wikelski 2002; Réale et al. 2010). This is because differences in fitness expectations can result in systematic differences in risk-taking behavior, including traits like aggressiveness, boldness, and exploration, that facilitate the trade-off between current and future reproduction: individuals with high future expectations, and thus much to lose should display risk-adverse behaviors while those with low expectations should invest into current reproduction through risk-prone behavior (Wolf et al. 2007; Réale et al. 2010). Although sex differences have not received much focus in the POLS framework, theories on life history variation, sexual selection, and sexual conflicts suggest that sex differences in the optimal life history strategy also commonly lead to predictable variation along the axis of pace-of-life (Wedell et al. 2006; Maklakov and Lummaa 2013; Adler and Bonduriansky 2014), and these can covary with behavioral differences (Hämäläinen et al. 2018, topical collection on Pace-of-life syndromes). As a consequence, sex differences may even generate POLS at the population level (Fig. S1).

Sex-specific optima for reproductive investment and life history scheduling are a result of a difference in the potential rate of reproduction between the sexes that ultimately stem from anisogamy (Bateman 1948; Maynard Smith 1982; Wedell et al. 2006; Lehtonen et al. 2016). The sex with the higher potential reproductive rate (typically males) (Andersson 1994), and/or a lower expected potential for future reproduction, is predicted to trade off self-maintenance against reproduction—including engaging in more aggressive and risky behaviors, while the slower-reproducing sex (typically females) with a higher potential for future reproduction is thought to experience stronger selection on self-maintenance. Sex differences in the reproductive costs, behavior, and physiology can result in differences in mortality risk and aging between males and females, which themselves provide feedback to sex-specific selection on life histories (Vinogradov 1998; Bonduriansky et al. 2008; Adler and Bonduriansky 2014). Sexual dimorphism in pace-of-life has been clearly demonstrated (e.g., Bonduriansky et al. 2008; Adler and Bonduriansky 2014), and there is also some evidence of systematic sex differences in POLS, whereby males were shown to be the faster sex across traits (e.g., Lovlie et al. 2014; Berger et al. 2014a, b, 2016). It is important to recognize, however, that the ways in which natural and sexual selection operate on each sex depends on the reproductive roles and mating system, breeding strategy, and environment, which influence whether selection is concordant or antagonistic between the sexes. Whether, and in which POLS traits, sex differences evolve is therefore predicted to differ across species, and potentially between populations. We discuss these tenets in our accompanying paper by Hämäläinen et al. 2018, topical collection on Pace-of-life syndromes (see also Tarka et al. 2018, topical collection on Pace-of-life syndromes).

Most studies on POLS have thus far focused on phenotypic correlations between traits, and without taking into account the potential for sex-specific POLS. However, expanding the approach to consider the genetic basis of POLS in each sex is necessary for answering several important evolutionary questions. The concept of POLS—i.e., covariation between behavioral syndromes and life history trade-offs—itself begs the question of how such a covariation is generated and whether it can evolve. The answer requires knowledge of the genetic architecture of POLS in each sex, in addition to understanding how selection operates on the sexes. Knowledge of the underlying genes also helps to understand how variation in natural populations is mechanistically created, and whether the same genes impact POLS within and across species. Identifying candidate genes can also reveal new traits not considered previously that may be important for POLS evolution through shared mechanistic basis.

Here, we bring together central insights from quantitative and molecular genetics and genomics to discuss how sexual dimorphism can evolve in POLS, owing to the sex-specific genetic architecture that arises from sex differences in gene expression and sex chromosome linkage. We present examples of important neuroendocrine pathways underlying animal personalities and highlight some of their pleiotropic effects on life history and physiological traits, with the aim to identify candidate genes and molecular pathways for future studies to test for the genetic integration underlying POLS. We similarly review the key mechanisms underlying life history trade-offs and discuss the roles of sex steroids in vertebrates and juvenile hormone in insects as regulators of sex differences in POLS. We also discuss the importance of considering morphology as an integral part of POLS due to many mechanistic links between POLS phenotypes and, e.g., body size or color variation. Although not exhaustive, our review aims at covering a broad range of potential mechanisms, to spark ideas and create a foundation for future research directions investigating the role of sex in the evolution of POLS.

## Genetic architecture of sex differences

### Evolution of multitrait dimorphism in the face of a shared genome

How males and females can evolve differences in the POLS traits is an important theoretical and empirical question. Evolution of sexual dimorphism is complicated by the fact that males and females of all species share the same genes, apart from those residing in the heteromorphic sex chromosomes, such as Y and W. Most homologous traits in the sexes will therefore have a shared genetic basis. Sexually antagonistic selection over optimal expression of a shared locus creates intra-locus sexual conflict (Parker 1979; Rice and Chippindale 2001; Arnqvist and Rowe 2005; Bonduriansky and Chenoweth 2009; Connallon and Clark 2014). This conflict can be resolved or at least mitigated by the evolution of sexual dimorphism, moving one or both sexes closer to their sex-specific adaptive optima (Lande 1980; Fairbairn et al. 2007; Bonduriansky and Chenoweth 2009; Connallon and Clark 2014; Connallon and Clark 2010). The amount of sex-specific genetic variance and the strength of the genetic covariance between the sexes will influence how readily sexual dimorphism can evolve (Lande 1980). Many homologous (and even non-homologous) traits show a strong positive genetic correlation between males and females suggesting that genetic constraints preventing phenotypic sexual divergence in the face of sexually antagonistic selection are common (Cox and Calsbeek 2009; Poissant et al. 2009).

Before turning to the genomic and molecular underpinnings of how the sexes may break the genetic correlation, we will discuss the complexity that necessarily accompanies evolution of POLS—genetic covariances between traits. Sexual dimorphism in POLS traits evolves largely as a result of sex-specific selection on single traits and/or on their correlations (Wyman et al. 2013; see Hämäläinen et al. 2018, topical collection on Pace-of-life syndromes for sources of sex-specific selection on POLS). Trait correlations can be created and altered in strength and directionality by correlational selection (e.g., Cheverud 1984; Kingsolver and Wiernasz 1987; Brodie 1989, 1992; Lynch and Walsh 1998; Sinervo et al. 2000; Svensson et al. 2001). Genetic trait correlations can be formed by pleiotropy or physical linkage, but adaptive combinations of alleles that would otherwise be broken down by recombination can be maintained by strong correlational selection even in unlinked loci. Frequency-dependent correlational selection can act to maintain variation in multitrait phenotypes (Sinervo and Svensson 2002) such as POLS. If (correlational) selection on POLS traits is sex-specific, this can result in sex differences in within-sex covariances between the traits—in strength and orientation—(Fig. S1), potentially even without a change in the trait means between the sexes. It may even be possible that POLS exists only in one sex, or that completely different traits form the syndrome in each sex due to fundamentally different trade-offs (Hämäläinen et al. 2018, topical collection on Pace-of-life syndromes). One important consequence of sex differences in the means along the axes of life history, behavioral, and physiological traits is that they can form a positive covariation between these traits across the sexes: sex may be the main cause for POLS at the population level (Fig. S1, see also Hämäläinen et al. 2018, topical collection on Pace-of-life syndromes).

Recognizing the two different levels at which variation in POLS traits can occur—*within* and *between* the sexes—is important because they may or may not align (Fig. S1). Consistent trait covariances between and within the sexes could arise due to the sexes evolving along the same trajectory as the trait covariances within sexes (for an analogous mechanism proposed at population level see Sokal 1978; Scheiner and Istock 1991). This is perhaps the most likely scenario given that sex-specific covariances can be constrained on multiple levels; by intra-locus conflict, just like evolution of mean differences, but also by physiological, developmental, and genetic constraints that govern trade-offs underlying the patterns of trait covariances, which may be harder to break by selection on one sex alone. Indeed, phenotypic traits are not varying as separate units, but are integrated in trait networks through genetic, developmental, physiological, and functional interactions (Arnold 1992; Armbruster et al. 2014), forming the conceptual basis for POLS theory (Ricklefs and Wikelski 2002; Réale et al. 2010). Although substantially more intricate, a multivariate view more closely reflects the true biological complexity of the genetic architecture and evolution of phenotypes (Walsh and Blows 2009), and also the evolutionary dynamics of multivariate sexual dimorphism (Lande 1980; Wyman et al. 2013). Therefore, evolution of multitrait phenotypes depends not only on the amount of additive genetic variance but also on the strength and directionality of additive genetic covariances between traits (together called the genetic (co)variance matrix or the G-matrix (e.g., Lynch and Walsh 1998) and the strength and directionality of multivariate selection acting on the G-matrix (Lande and Arnold 1983). The direction of selection will matter, because there might not be equal amount of additive genetic variance in all directions of the multivariate character space, restricting the directions in which traits and trait combinations can respond to selection, i.e., evolve (Schluter 1996; Hansen and Houle 2008; for visualization see Fig. 1 in Teplitsky et al. 2014).

**Fig. 1:**
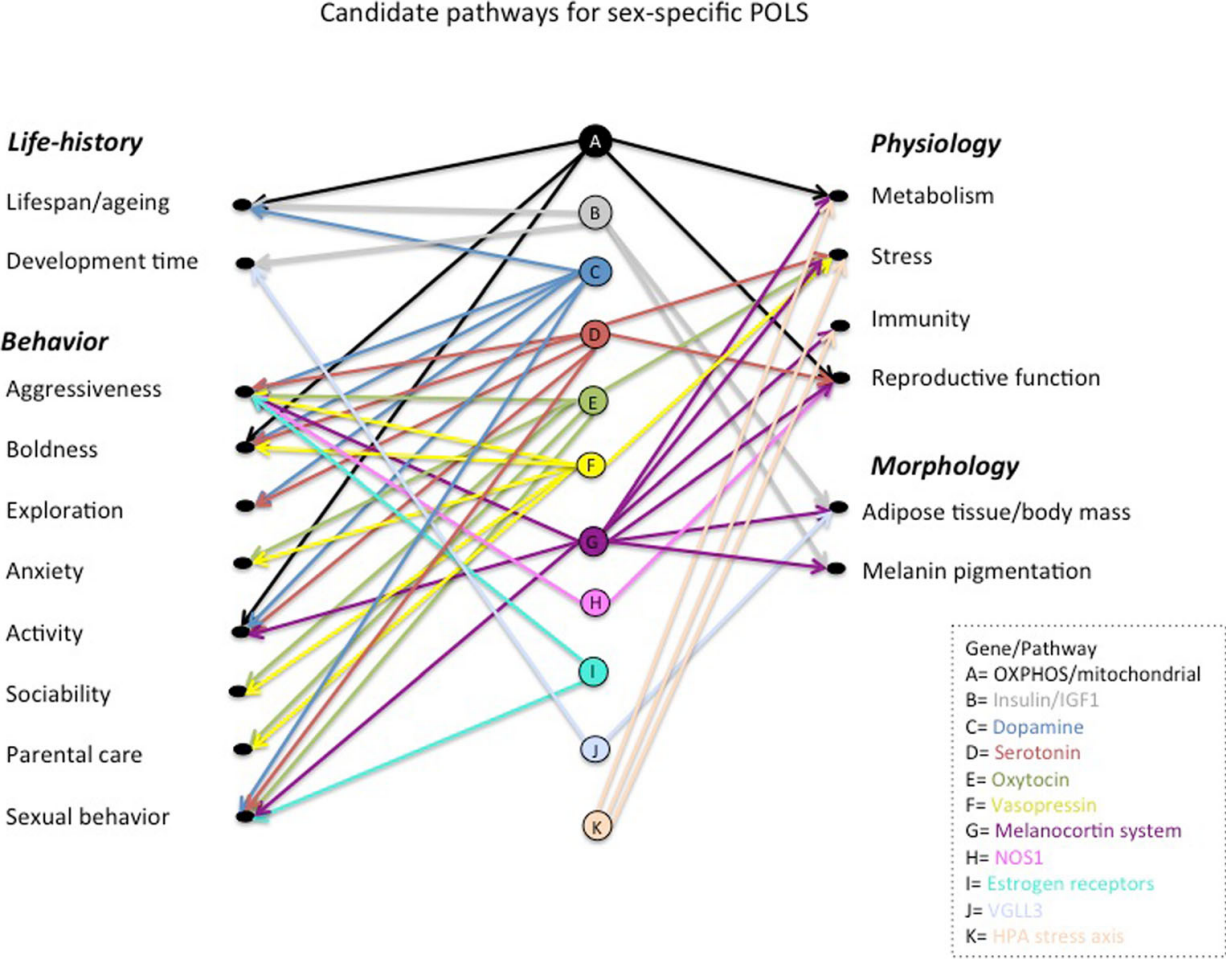
Examples of candidate genes and molecular pathways (highlighted with different letters and colors) that influence multiple traits associated with POLS, with evidence for sex specificity in gene action and/or function. See Tables 1 and 2 for species, description of effects, and references

Formally, the G-matrix can be broken down into sex-specific G-matrices (G_male_ and G_female_), each consisting of genetic variances and covariances of traits within each sex, and the cross-sex genetic trait covariances (called the B-matrix) (Lande 1980; Reeve and Fairbairn 1996; Gosden et al. 2012; Wyman et al. 2013). Both the sex-specific G-matrices and the B-matrix will together influence the speed and direction of the evolutionary response to multivariate selection in each sex (Lande 1980; Gosden et al. 2012; Wyman et al. 2013). Strong, positive cross-sex genetic covariances between traits (i.e., in the B-matrix) will mainly constrain evolution of mean differences *between* sexes and strong, positive trait covariances within sexes (i.e., the G_male_ and G_female_) will constrain changes in the trait means *within* sexes. Recent studies exemplify these tenets in terms of the evolution POLS in a seed beetle *Callosobruchus maculatus*, where sexually antagonistic selection operates on an integrated phenotype (metabolic rate, lifespan, behavioral activity and body weight) favoring a “fast” life history strategy in males and a “slow” in females. Artificial male-limited selection on lifespan revealed an intra-locus sexual conflict due to genetic correlations between the traits within and between the sexes. In addition to these constraints, evolutionary response in activity levels in the direction of male-specific selection (i.e., for higher levels) was limited by lack of sufficient additive genetic variance with consequences on sex differences in POLS trait covariance (Berg and Maklakov 2012; Berger et al. 2014a, b). The cross-sex covariances can even cause evolutionary response in the opposite direction to that of selection: In great reed warblers (*Acrocephalus arundinaceus*), for example, wing length is under sexually antagonistic selection and despite higher fitness in females with shorter wings, female wing length is expected to increase due to a correlated response to selection in males (Tarka et al. 2014).

Given these constraints, how can sexual dimorphism be so commonplace and how do we expect it to evolve in POLS? There are a number of mechanisms that may facilitate the process. Tightly integrated traits, as predicted by the POLS framework, can more readily respond to selection as a unit, so that when selection acts on one trait, a correlated response is obtained in coadapted traits (Futuyma 2010). However, this alone does not circumvent the putative constraint from strong genetic covariances between the sexes (Poissant et al. 2009; Gosden et al. 2012). It should be noted that constraints are not usually absolute, and as long as the genetic correlation deviates from unity and there is sufficient genetic variation for the trait, sexual dimorphism will evolve, albeit slowly. Another way to circumvent constrains from the genetic correlation between sexes is through sex differences in the amount of additive genetic variance (e.g., found for aggression and exploration in crickets: Han and Dingemanse 2017). Even when under the same strength and direction of selection, the sex with more additive genetic variance will evolve faster than the sex with less additive genetic variance (even if the genetic correlation is 1), leading to evolution of dimorphism (Lande 1980; Cheverud 1984; Wyman and Rowe 2014). This suggests that in addition to sources of sex-specific selection, mechanisms that lower the cross-sex covariance and create differences in the additive genetic variances are important for the evolution of sexual dimorphism in POLS.

Whether the genetic architecture will pose constraints on evolution also depends on how stable it is over time. The stability of the G-matrix in time and space is an ongoing debate, with some studies supporting conserved matrices between populations and environments (e.g., Delahaie et al. 2017) and temporal stability (e.g., Garant et al. 2008), while others show rapid fluctuations over time (e.g., Björklund et al. 2013). Variation in the strength of genetic covariances enables faster independent evolution of traits within or between sexes during the times when covariances are lowered. The within-sex genetic (co)variance matrix (G_male_ and G_female_) is considered to be more stable over time than the cross-sex covariance matrix (B), which suggests that there is more constraint to change POLS trait covariances within the sexes than it is between them (Barker et al. 2010; Gosden and Chenoweth 2014). Low stability in the B-matrix should open up periods of time with possibilities of independent evolution of the sexes, generating differences in means and covariance patterns between sexes. Artificial selection experiments have shown that both differences in means (Alicchio and Palenzona 1971; Bird and Schaffer 1972) and cross-sex genetic correlations can be quite readily changed (Delph et al. 2011). Nevertheless, several studies show that the cross-sex genetic correlation constrains evolution within (Arnqvist and Tuda 2010), and across species (Schluter 1996; Poissant et al. 2009), suggesting that at least some stability in the genetic architecture across sexes persists over evolutionary time scales. Further investigation is therefore clearly needed on the stability of G and B-matrices.

These insights suggest that sex differences in POLS may evolve even in the absence of sex-specific selection, but become especially likely in its presence affecting both trait means and covariances. However, we are currently lacking studies of multivariate G- and B-matrix architecture of POLS traits in both sexes, which are essential in predicting the evolutionary trajectories of POLS. Experimental evolution or artificial selection studies can be useful for quantifying to what extent genetic architectures are constraining contemporary evolution of POLS in the sexes, while studying the G- and B-matrices in a comparative context would help to understand how species vary in the genetic integration of the different POLS traits and to what extent this is sex-dependent and predictable based on the mating system or other factors that affect sex-specific selection regimes (Hämäläinen et al. 2018, topical collection on Pace-of-life syndromes). G- and B-matrices can be estimated using animal models with pedigreed wild populations or by laboratory breeding designs, including using artificial selection or experimental evolution lines. It is important to note, however, that reliable estimation of covariance matrices requires a lot of data because of the typically large sampling variance, which makes this line of work labor-intensive and subsequently costly, and perhaps most suitable for species with relatively shorter generation times and possibility for laboratory breeding.

### Genomic location for genes with sex-specific effects

Where in the genome can we predict to find loci encoding sexually dimorphic POLS phenotypes? This question matters because the genomic location determines in part the genetic architecture of variation in POLS in each sex. Sex chromosomes are subject to different dynamics of sex-specific selection because, unlike autosomes, they are unevenly inherited between males and females. Because they are the only regions of the genome that differ between the sexes, much research has been devoted to understanding what part do sex chromosomes play in accumulating loci with sexually antagonistic effects. Indeed, under some conditions, sex chromosomes can become hot spots for sexually antagonistic genes (Rice 1984; Albert and Otto 2005; Connallon and Clark 2010; Fry 2010). Importantly, this theory could be expanded to sexual dimorphism, which may evolve when modifiers to the antagonistic loci arise in their vicinity that allow sex-specific expression of the antagonistic loci, and hence the development of sexual dimorphism in the phenotype (Rice 1984; but see Connallon and Clark 2010).

The sex chromosomes X and Z  may also contribute disproportionately to the within-sex genetic variation in a sexually dimorphic trait in the heterogametic sex (Reinhold and Engqvist 2013). This idea is based on the logic that genetic variance in the homogametic sex can be reduced by heterozygosis, whereas in the heterogametic sex the allelic effects are immediately exposed (Reinhold and Engqvist 2013). In support of these predictions, Reinhold and Engqvist (2013) found that genetic variance in body size is greater in males of X/Y species and in females of Z/W species. The heterogametic sex could therefore generally show greater amounts of additive genetic variance in POLS traits associated with the X/Z chromosomes than the homogametic sex. How this contributes to the total amount of additive genetic variance will also depend on dosage compensation and the contribution of autosomal alleles (Wyman and Rowe 2014).

Sex differences in recombination rate (i.e., heterochiasmy) is also expected to influence the genomic distribution of loci under sex-specific selection (Connallon and Clark 2010; Wyman and Wyman 2013). Heterochiasmy is very common across taxa and can facilitate coinheritance of beneficial alleles across loci, such as those influencing correlations between POLS traits, in a single linkage group in one sex while allowing their reshuffling in the other (Lenormand 2003). The genomic locations that show heterochiasmy differ among species, and while they also occur in autosomes, the chromosome with sex-determining locus is the most famous such location (Ritz et al. 2017). Linkage with this locus is a safe zone for loci with sexually antagonistic effects because of the reduced or absent recombination (Wright et al. 2017). Y-chromosome (which contains the sex-determining locus in *Drosophila*) for example influences the expression of many autosomal and X-linked male-biased genes with roles in male reproduction and metabolism, despite having only few genes (Lemos et al. 2008). Y-linked expression modifiers could therefore indirectly regulate other POLS traits that depend on metabolic pathways in males (see also below).

Taken together, there are many circumstances where sex chromosomes are expected to play a disproportionate role in harboring loci for sexually dimorphic traits, and empirical evidence provides support for this (Mank 2009; Dean and Mank 2014; Mank et al. 2014; but see, e.g., Husby et al. 2013), even for behavior and brain sexual differentiation (De Vries et al. 2002; Arnold et al. 2004; Gatewood et al. 2006). However, the exact conditions under which sexual dimorphism evolves via sex-linked loci are still debated (Rice 1984; Connallon and Clark 2010; Fry 2010) and autosomes could also be important (Fry 2010). Future studies that investigate the chromosomal linkage patterns of POLS traits in both sexes, combined with a detailed understanding of how trait variation influences fitness in each sex, will help to elucidate where in the genome POLS variation is encoded. Narrowing down the candidate genes will further allow identifying the molecular mechanisms of sexual dimorphism, such as sex-specific regulation of gene expression, tissue-specificity of such patterns, and putative pleiotropic effects on multiple POLS traits.

### Sex differences in gene expression underlie sexually dimorphic phenotypes

At the molecular level, phenotypic differences between the sexes should arise through differential expression of the shared genes, evolved in response to sex-specific selection to resolve the intra-locus sexual conflict (Ellegren and Parsch 2007; Mank 2017). The evidence for these straightforward predictions is accumulating but remains anecdotal (Hollis et al. 2014; Immonen et al. 2014; Harrison et al. 2015; Cheng and Kirkpatrick 2016; Veltsos et al. 2017). Expression differences occur in a large proportion of animal genomes and can be influenced for example by gene duplication followed by evolution of sex-specific gene expression of the new paralog (Connallon and Clark 2011), as well as genomic imprinting (Iwasa and Pomiankowski 2001). Genes with a higher expression in one sex are generally thought to play a more important functional role and experience stronger selection in that sex (e.g., Ellegren and Parsch 2007), and particularly male-biased genes show elevated rates of both gene expression and protein sequence divergence between species (Ellegren and Parsch 2007; Grath and Parsch 2016). However, the patterns of sex-biased expression can be specific to each tissue (Grath and Parsch 2016), developmental (Perry et al. 2014) and reproductive status (Immonen et al. 2017), and therefore, the sex-bias status of the gene may not always be a reliable indicator of its fitness consequences (see also Mank 2017).

In principle, sex-biased expression results from ontogenic sexual differentiation by sex determination cascades. In genetic sex determination, which is the most common mechanism in metazoans, the signal that induces sex differentiation via triggering the development of the gonads is carried by sex chromosomes (Saccone et al. 2002; Smith and Sinclair 2004; Jazin and Cahill 2010). Subsequent sex differences in many somatic traits results from sex-specific alternative splicing that regulates sex-biased expression in a tissue- and cell-specific way (Hartmann et al. 2011).

Although the initial sex differentiation arises from sex-linked genes independent of hormonal control (Arnold 2009), in vertebrates, sex steroids are regarded as the master regulators of most sex differences (Zauner et al. 2003; Ketterson et al. 2005; Mank et al. 2007), including in POLS (see below). In line with this, a recent study in the brown anoles lizard (*Anolis sagrei*) showed how testosterone regulates changes in sex-biased expression in genes involved in growth and metabolism associated with the development of sexual size dimorphism (Cox et al. 2017). Testosterone-mediated changes in sex-biased expression are also likely behind male-limited polymorphism in life history strategies in the wild turkey (*Meleagris gallopavo*), in which sexual dimorphism in the gene expression correlates with dimorphism in behavior and plumage (Pointer et al. 2013).

There has been much progress in identifying sex hormone receptor molecules and their sex-dependent expression patterns (Reinius et al. 2008; Wu and Shah 2011). Estrogen signaling is primarily conveyed by the estrogen receptors alpha and beta (ERα and ERβ) that bind to specific DNA sequences—estrogen-responsive elements (EREs)—resulting in the transcriptional activation of genes in sex-specific ways (Jazin and Cahill 2010). ERα and ERβ are particularly interesting for animal “personalities” through their regulatory effect on the oxytocin pathway (see Table 1, Fig. 1). Also androgens exert their function by binding to androgen receptors, which in turn cause the expression of target genes by binding to different sets of androgen-responsive elements (Jazin and Cahill 2010). Overall less is known about the downstream targets of sex hormone receptors and how specific variation in behaviors are regulated in the sexes. A study in mice (*Mus musculus*) suggests that the different features of male sexual behavior, male aggression, maternal behavior, and female sexual behavior are controlled by many sex-specifically regulated genes (including *Brs3*, *Cckar*, *Irs4*, *Sytl4*) with highly specific effects on components of behaviors, rather than few genes with broad pleiotropic effects on whole behaviors (Xu et al. 2012). Such architecture may allow selection to fine tune behaviors in the sexes more easily without disruption of complete behaviors.

**Table 1:** Candidate molecules influencing behavioral syndromes; their putative pleiotropic effects on life history and physiological traits and evidence for sex differences in activity or function. The superscript numbers connect a focal trait with an example of a species and reference where the effect has been observed (separately for each molecule/pathway)

Molecule/pathway	Function	Affected traits	Organism	Sex-specific effect	References*
Dopamine receptors: D4 (DRD4)	Receptor for dopamine neurotransmitter in the dopaminergic system	Activity/impulsiveness/ADHD/restlessness^1^ Novelty seeking^2^ Boldness (risk-taking)/fearfulness^3^ Lifespan^4^ Sexual behavior/function^5^ Exploration^6^ Neophobia^7^ Aggression^8^	Humans^1,2,3,4,5^ Rhesus macaques (*Macaca mulatta*)^1,3,6^ Mice (*Mus musculus*)^1,4^ Rats (*Rattus norwegicus*)^5^ Dogs (*Canis lupus familiaris*)^1^ Dunnock (*Prunella modularis*)^3,5^ Great tits (*Parus major*)^3,6^ Yellow-crowned bishop (*Euplectes afer*)^6^ Collared flycatchers (*Ficedula albicollis*)^3,7,8^ Black swans (*Cygnus atratus*)^3^	In humans, association with novelty seeking and aggressive impulsiveness stronger in males and dependent on environment. Stronger association with lifespan in females. No reported sex difference in the association with sexual behavior. In macaques, sex-specific effects not tested. In mice, sex not reported and sex-specific effects not tested. Only male mice and rats tested for hyperactivity and sexual function defects, respectively. No sex difference in activity and impulsiveness in dogs. Multiple alleles with sex-specific associations with breeding strategy and boldness/risk-taking behavior in dunnocks. In great tits, no sex-specific effects found on exploration, but there is an association with fearfulness (in the context of parental provisioning) only in males. No sex difference found in exploration in the yellow-crowned bishops. In collared flycatchers, effects in males (in the context of territorial defense, no females were studied). In black swans, sex not reported and sex-specific effects not tested for boldness.	Faraone et al. 2001^1^; Schinka et al. 2002^2^; Grady et al. 2005^1^, 2013^4^; Avale et al. 2004^1^; Melis et al. 2006^5^; Li et al. 2006^1^; Ben Zion et al. 2006^5^; Heijas et al. 2007^1^; Fidler et al. 2007^6^; Munafò et al. 2008^2^; Korsten et al. 2010^6^; Dmitrieva 2011^1^; Carpenter et al. 2011^3^; Schilling et al. 2014^11^; Mueller et al. 2014^6,7^; Garamszegi et al. 2014^3,7,8^; Coyne et al. 2015^1,3,6^; Timm et al. 2015^3^; Van Dongen et al. 2015^3^; Holtmann et al. 2016^3,5^
D1 (DRD1) and D2 (DRD2)	Receptors for dopamine neurotransmitter in the dopaminergic system	Aggression	Humans and lab mice	Commonly, only males tested.	Reviewed in Nelson and Trainer 2007
Serotonin transporter (SERT or 5-HTT)	Monoamine protein that transports serotonin from the synaptic clefts to the presynaptic neurons, and therefore responsible for recycling of serotonin	Sexual behavior/function^1^ Anxiety^2^ Hyperactivity/ADHD^3^ Stress-aggression interaction^4^ Adaptation to urban life^5^ Risk taking^6^ Neophobia/neophilia^7^	Humans, lab mice, lab rats^1,2,3,4^ Blackbirds (*Turdus merula*)^5^ Great tits^5^ Dunnocks^1,6^	Rat studies show sex-shared effects of SERT. SERT-dependent, sex-specific effect of stress on aggression. In studies for adaptation to urban life, sex not reported and sex-specific effects not tested. Sex-specific associations on both risk taking and breeding strategy in multiple alleles.	Hull et al. 2004^1^; Serretti et al. 2006^2^; Verona et al. 2006^4^; Olivier et al. 2010^1^; Chan et al. 2011^1^; Mueller et al. 2013^5^; Jannini et al. 2015^1^; Riyahi et al. 2015^5,7^; Holtmann et al. 2016^1,6^
Serotonin (5-HT) and its receptors 5-HT_1a_ and 5-HT_1b_		Aggression^1^ Sexual behaviors/function^2^	Lab mice^1^ Humans^1^ Lab rats^2^	Differential 5-HT_1a_ binding potential may contribute to the sex difference in aggression. Sexually antagonistic effects of serotonin receptor 5-HT_1A_ on sexual behaviors in rats.	Parsey et al. 2002^1^; (review) Nelson and Trainer 2007^1^; Olivier et al. 2010^2^
Monoamine oxidase A (MAOA)	Catabolizes oxidative deamination of neurotransmitters dopamine, norepinephrine, and serotonin	Aggression	Humans Lab mice	Association studied in males, and in humans, MAOA variation effects interact with early environment.	Brunner et al. 1993; Shih et al. 1999; Manuck et al. 2000; Beitchman et al. 2004; Frazzetto et al. 2007; Kim-Cohen et al. 2006; Scott et al. 2008; MacDermot et al. 2009
Nitric oxide synthase 1 (NOS1)	One of several genes responsible for synthesis of nitric oxide, which performs many neurotransmitter functions	Aggression Sexual function	Lab mice	Effects in males in interaction with testosterone	Nelson et al. 1995; Kriegsfeld et al. 1997; (review) Nelson and Trainer 2007; Trainor et al. 2007
Estrogen receptor α (ERα) and β (ERβ)	A transcription factor activated by the sex hormone estrogen	Territorial aggression^1^ Parental care^2^ Female preference^3^ Aggression^4^ Anxiety^5^	White-throated sparrows (*Zonotrichia albicollis*)^1,2^ Prairie voles (*Microtus ochrogaster*) ^2,3^ Beach mice (*Peromyscus polionotus*)^4,5^ Rats^5^ Mice^4^	ERα: association with parental care variation only in males (in prairie voles only males studied). Both ERα and ERβ affect aggression in males, depending on the photoperiod (only males studied in beach mice). In rats, anxiety (and fear-induced learning) is female-specific with opposite effects of ERα and ERβ.	Scordalakes et al. 2003^4^; Horton et al. 2014^1,2^; Cushing et al. 2008^2,3^; Trainor et al. 2007^4,5^; Toufexis et al. 2007^5^
Oxytocin (OXT) and its receptor (OTR)	Oxytocin is both a non-neural hormone and a neuropeptide.	Partner preference^1^ Pair-bonding^2^ Mate guarding^3^ Social bonding and recognition^4^ Parental care^5^ Aggression^6^ Stress^7^ Anxiety^8^	Voles (*Microtus*^1,2,3,4,5,6^, *Lasiopodomys*) Rats^4^ Mice (*Peromyscus*^7^, *Mus*^8^, *Scotinomys*) Hamsters^6^ (*Cricetulus*) Mongolian gerbils (*Meriones unguiculatus*) Social tuco-tuco (*Ctenomys sociabilis*) Humans^5,7^ Zebra finches^2,4,6,7^ (*Taenioypygia guttata*)	Sex-differences in brain region-specific activity of OXT and OTR binding are common (genus/species listed) and often species-specific. Effects are estrogen and androgen modulation. Behaviors are often affected differently in the sexes in humans and rodents.	(review) Heinrich and Domes 2008^4,6,7,8^; Guzman et al. 2013^8^; Kelly and Goodson 2013^2,4,6^; Holley et al. 2015^3^; (review) Dumais and Veenema 2016^1,2,4,5^; Steinman et al. 2016^7^; Li et al. 2016^8^; (review) Feldman and Bakermans-Kranenburg 2017^5^
Vasopressin (VP) and its receptors (V1R with subtypes a and b, V2R)	Vasopressin is both a non-neural hormone and a neuropeptide.	Partner preference^1^ Pair-bonding^2^ Social bonding and recognition^3^, aggression^4^ Prepulse inhibition^5^, Dominance^6^ Parental care^7^ Stress^8^ Anxiety^9^	Voles (*Microtus*^1^, *Lasiopodomys*) Rats^3^ Mice (*Peromyscus*^7^, *Mus*^9^, *Scotinomys*, *Eliomys*) Hamsters^4^ (*Cricetulus*) Mongolian gerbils (*Meriones unguiculatus*) Social tuco-tuco (*Ctenomys sociabilis*) Humans^2,5^ Marmosets (*Callithrix jacchus*) Chimpanzees^6^ (*Pan troglodytes*) Zebra finches^2,3,4,8^ (*Taenioypygia guttata*)	Sex-differences in brain region-specific activity of VP and V1aR binding are common (genus/species listed) but often species-specific. Effects are estrogen and androgen modulation, and sensitive to changes in dominance status, season, and photoperiod. Behaviors are often affected differently in the sexes (examples numbered) in the rodents and humans where these have been studied (but sexes are rarely studied simultaneously). For example, V1aR affects anxiety and social recognition only in male mice.	Bielsky et al. 2005^3,9^; (review) Kelly and Goodson 2013^2,3,4^; Albers 2015; (review) Dumais and Veenema 2016^1,2,4,5,6,10,11^; Bendesky et al. 2017^7^
Melanocortin system: melanocortins (α-, β-, ϒ-MSH, ACTH), encoded by *POMC*, and their receptors MC1-5R and their antagonists (ASIP and agouti-related protein, AGPR).	Melanocortin system consists of melanocortin peptides α, β, and ϒ-melanocyte-stimulating hormone (α-, β-, ϒ-MSH), and drenocorticotropic hormone (ACTH) (encoded by a prohormone gene *POMC* through posttranslational modifications). The G-coupled melanocortin receptors include MC1R and four others (MC2-5R). Agouti-related protein (AGPR) is an antagonist at MC3R and an inverse agonist and antagonist at MC4R. ASIP is its homolog in the skin. α-MSH also interacts with oxytocin and dopamine via MC4R.	*Sexual behavior*^1^: Pair-bonding, female sexual receptivity, male sexual motivation and fertility, testosterone, progesterone, FSH and GnRH hormone levels (MC2R, MC4R) *Aggression*^2^: Aggressiveness and exocrine gland activity (MC5R) *Energy homeostasis*^3^: for example, physical activity, food intake, thyroid hormone activity, metabolic rate, adult adipose tissue (MC3R, MC4R) *Stress response*^4^: anxiety, glucocorticoid plasma and basal adrenocortocotropic levels, resistance to stressors (MC2R, MC4R), grooming, stretching, and yawning *Immune function*^5^: mechanisms of anti-inflammation (MC3R, MC4R, MC5R)	Lab mice^1–5^ Eleonora’s falcon (*Falco eleonorae*)^4^ Dogs^3^ Humans^1,3,5,6^ Kestrel (*Falco tinnunculus*)^3,5^ Prairie voles^1,2,4^	Melanocortins affect sex hormone production^1^, with putative consequences on sexual dimorphism. However, sex specificity has rarely been tested, and most studies include only one sex (e.g., males in studies of aggressiveness) and many do not report the sex of the subjects. Sex differences in the genetic correlations between immunity, body mass, and melanic coloration demonstrated in kestrels. Sex differences in adult pair-bonding and juvenile social behaviors (aggression and exploration) were observed in prairie voles, associated with MC4R activity via activation of oxytocin, vasopressin neurons. No difference in the effect on corticosterone levels between the sexes. Sex differences in energy expenditure and food intake in mice associated with MC3R and MC4R activity, possibly due to female-biased expression of AGPR. Male-limited inflammatory response in Eleonora’s falcon associated with color and *MC1R* polymorphism.	(review) Gantz and Fong 2002; (review) Chaki and Okuyama 2005^5^; Goodin et al. 2008^3^; (review) Ducrest et al. 2008^all listed traits^; Gangoso et al. 2011^4^; Kim et al. 2013^3,5^; Barrett et al. 2014^1,2,4^; Lensing et al. 2016^3^

*See the supplementary file for references

## Mechanisms for POLS

Most traits have a highly polygenic basis, and empirical evidence suggests that this may also be true for trait correlations (Saltz et al. 2017). Both linkage disequilibrium (LD), due to correlational selection or physical linkage, and pleiotropy can underlie genetic correlations forming POLS (these are not mutually exclusive; Saltz et al. 2017). LD solely due to correlational selection on unlinked loci is considered evolutionarily less stable due to recombination; however, LD formed by large physical rearrangements such as inversions are a powerful (although relatively rare) way of forming trait correlations, because they can capture multiple genes in a single non-recombining locus. Gene pleiotropy is however likely to be most relevant for POLS because of trade-offs that are at the heart of pace-of-life variation and covariation with behavioral syndromes (Réale et al. 2010). Trade-offs reflect functional relationships between traits that compete over finite resources, and therefore, any variant that influences investment in one trait must necessarily have pleiotropic consequences on other traits dependent on the same resource (Houle 1991). It is important to note, however, that there are many, mutually non-exclusive ways how to define pleiotropy (Paaby and Rockman 2013). For the purpose of this section (summarized in Fig. 1 and Tables 1 and2), we combine evidence of both “molecular gene pleiotropy” (i.e., evidence of functional pleiotropy from, e.g., gene knockout and pharmacological studies) and “developmental pleiotropy” (evidence of mutations influencing the phenotype-genotype map and ontogenic relationships of traits). Here, our aim is to present some of the most interesting candidate molecules involved in neural and physiological pathways that can and should be studied further to test if genetic variation and/or covariation between POLS traits maps to any of the genes involved that could explain for example sex-specific fitness variation or antagonistic pleiotropy between current and future reproduction or early and late life.

**Table 2:** Examples of candidate molecules and pathways influencing life history, morphological, and physiological traits involved in variation in the pace-of-life, their pleiotropic effects on other traits and evidence of sex differences in activity and/or function. The superscript numbers connect a focal trait with an example of a species and reference where the effect has been observed (separately for each molecule/pathway)

Molecule/pathway	Function	Affected traits	Organism	Sex-specific effect	References*
Vestigal-like family member 3 (VGLL3)	Cofactor for the TEA domain family of transcription factors involved in adiposity regulation.	Age at sexual maturity^1^ Size at sexual maturity^2^	Salmon (*Salmo salar*)^1^ Humans (*Homo sapiens*)^1,2^	In salmon, alternative alleles delay female and advance male age at puberty, with sex-specific dominance. In humans only, females were tested.	Cousminer et al. 2013; Barson et al. 2015; Ayllon et al. 2015
Insulin-like growth factor 1 (IGF1) signaling pathway	Insulin and IGF1 exert their effects by activating cell surface transmembrane receptors that phosphorylate a variety of substrates (including insulin receptor substrate, IRS, proteins). IRS activate several downstream cascades including mTOR pathways.	*Pace*-*of*-*life*^1^: growth (body mass), age at sexual maturity, and lifespan *Sexual signals*^2^: cuticular hydrocarbons, exaggerated weaponry	For example, 41 mammalian species (see Swanson and Dantzer 2013)^1^ Lab mice (*Mus musculus*)^1^ *Drosophila* fruitflies^1,2^ *Caenorhabditis* nematodes^1^ Rhinocerus beetles (*Trypoxylus dichotomus*)^2^	Sex-specific effects of insulin signaling and activities of IGF1 receptor and mTOR are well-documented for lifespan, growth, and body size in both mammals and insects. Sex-effects are also species-specific. Insulin/IGF signaling also affects condition dependent sexual ornamentation.	Emlen et al. 2012^2^; Kuo et al. 2012^2^; Swanson and Dantzer 2013; (review) Brooks and Garratt 2017^1,2^
Mitochondrial DNA (mtDNA) and epistatic interactions with nuclear-encoded genes.	37 mtDNA genes encode products that interact with a proteome encoded by nuclear genes to form an oxidative phosphorylation pathway (OXPHOS) pathway responsible for oxidizing nutrients to reform released energy into ATP. Other mitochondrial functions include apoptosis and ROS signaling.	Lifespan, ageing^1^ Reproduction^2^ Behaviors: locomotor activity, proactivity ^3^ Metabolic rate^4^	Seed beetles^1,2,3,4^ (*Callosobruchus maculatus*, *Acanthoscelides obtectus*) *Drosophila* fruitflies^1,2^ Bank voles (*Myodes glareolus*)	Variation in mtDNA and mitonuclear epistasis commonly have sex-specific effects on lifespan, aging, and reproduction-related traits. Sex-specific bioenergetics and metabolic rate associated with variation in the pace-of-life in a seed beetle that differ in mtDNA haplotypes. A male-limited correlation between metabolic rate and proactive behavior associated with mtDNA variation has been demonstrated in a bank vole.	Arnqvist et al. 2010^4^; Løvlie et al. 2014^1,3^; Sichova et al. 2014^3,4^; Dobler et al. 2014^1,2^; Camus et al. 2015^1,2^; Immonen et al. 2016a,b^2,4^; Arnqvist et al. 2017^1,2,3^
Genes encoding corticotropin-releasing factor (CRF), adrenocorticotropic hormone (ACTH), melanocortin-2 receptor (MC2R), genes involved in glucocorticoid synthesis, and the receptors for glucocorticoids (GR)	The hypothalamic-pituitary-adrenal (HPA) stress axis is driven by CRF neurons. CRF activation upon stress results in a release of ACTH into the general circulation to activate MC2R in the adrenal gland cortex, which activates the synthesis and release of glucocorticoids via binding to GR glucocorticoids exert diverse effects from stress and immunity to general homeostasis and development.	Stress Immunity Metabolism Cognition (memory) Homeostasis Development	Laboratoty mice (*Mus musculus*) Rats (*Rattus norwegicus*) Humans	Sex differences observed throughout the HPA axis in both lab rodent models and humans. Sexual dimorphism in the HPA develops via influence from testosterone and in interaction with environment (strong maternal effects).	(review) Bale and Epperson 2015

*References can be found in a supplementary file

Below, we summarize the candidate pathways for (I) behavioral syndromes and (II) life history trade-offs that have shared effects on multiple POLS traits, and discuss evidence for sex specificity in these effects (Fig. 1, Tables 1 and 2). We specifically discuss the roles of nutrient sensing pathways (“Nutrient sensing pathways”) and glucocorticosteroids (“Glucocorticoids”) in mediating life history trade-offs, as well as metabolic genes (“Metabolic genes”), with a focus on the evolutionary consequences of their inheritance patterns, and male seminal fluid proteins as mediators of life history trade-offs and behavior in females (“Male seminal fluid proteins affect female behavior and life-history trade-offs”). We also discuss the importance of sex steroids and juvenile hormone in mediating sex-specific effects in POLS (III), and lastly, we argue why body size should be considered as an integral part of POLS (IV).

### Mechanisms behind behavioral syndromes

In the POLS framework systematic differences in risk-taking behavior, including traits like aggressiveness, boldness, and exploration are expected to coevolve with the trade-off between current and future reproduction (Wolf et al. 2007; Réale et al. 2010). This implies that the neuroendocrine control of behaviors may be coupled with life history variation either indirectly (e.g., through increased mortality due to fighting), or directly, through shared functions, e.g., in metabolism, immunity, and/or reproductive capacity (Fig. 1). How such a network of effects may have evolved is beyond the scope of this article, but correlational selection to position the focal genes under a shared regulatory pathway (see, e.g., the melanocortin system below) has likely played a role. Gene pleiotropy is often discussed in the context of genetic constraints for future evolution; however, selection for alternative life history strategies could have favored such architecture as the most parsimonious way for phenotypic integration.

A growing body of work demonstrates that behavioral syndromes have a genetic basis (van Oers et al. 2005; Dochtermann 2011; Dochtermann et al. 2015; Han and Dingemanse 2017) and studies on humans and rodent models have identified several molecular pathways likely contributing to the genetic correlations among systematic behaviors. Whether the same behavioral pathways influence life history, variation has rarely been studied, but provides a promising future avenue of research. Sex differences have been found in most cases where these pathways have been studied; however, there is a striking bias in studies in favor of using only males as subjects or not testing sex-specific effects when both sexes are included (e.g., Dumais and Veenema 2016).

Perhaps the most promising candidate pathway for POLS in vertebrates is the melanocortin system, which regulates several behavioral traits (aggressiveness, activity and sexual behavior), physiology (metabolism, stress response via HPA axis, immunity and reproductive function) and also morphology (body mass via regulation of feeding rate and adipose tissue, melanin coloration) (Fig. 1, Table 1) (Ducrest et al. 2008; Mundy 2005; Roulin and Ducrest 2013). The melanocortin system is most famous for its effects on melanin pigmentation, which is often a sexual signal and under sex-specific selection (e.g., Saino et al. 2013). Although color variation is not part of the POLS concept, the melanin polymorphism correlates remarkably well with POLS: darker individuals across taxa are often more aggressive (e.g., Mafli et al. 2011), have higher metabolic rate, physical and sexual activity levels (Ducrest et al. 2008), and show increased exploratory behavior (Mateos-Gonzalez and Senar 2012), compatible with a faster pace-of-life syndrome (but see, e.g., Kittilsen et al. 2009 that shows dark individuals have higher resistance to stressors). Sex specificity in the effects has rarely been studied, but melanocortins affect sex hormone production, through which its effects could be sex-specifically modulated. In kestrels (*Falco tinnunculus*), there is a sex-specific genetic correlation between melanic coloration, immunity, and body mass (Kim et al. 2013) implying sex differences in the melanocortin pathway. Melanin variation is also differently associated with life history strategies in the sexes (Emaresi et al. 2014; Meunier et al. 2010). Melanocortin system consists of a gene *POMC* that encodes for multiple melanocortin hormones that bind to different receptors with downstream effects on a great variety of traits (Table 1) (Cone 2005). One of the receptors, MC4R, has been implicated to affect sexual (pair-bonding), aggressive, and exploratory behaviors differently in the sexes in prairie voles (*Microtus ochrogaster*), while its effects on corticosterone levels are sexually monomorphic (Barrett et al. 2014). In laboratory mice, the receptors MC3R and MC4R affect energy expenditure and foraging differently in the sexes, possibly due to female-biased expression of agouti-related protein (AgRP), which is an antagonist for both of these receptors (Lensing et al. 2016). Because of the staggering array of effects relevant to POLS (Cone 2005; Ducrest et al. 2008) that link both behavioral variation and physiological traits important for life history trade-offs, the melanocortin system is an interesting candidate pathway even in species that lack melanin polymorphism.

Animal “personality” variation is regulated by neurotransmitters dopamine and serotonin systems, which affect various behaviors related to risk-taking and activity with some evidence for sex-specific effects (Table 1, Fig. 1). The gene-encoding Dopamine receptor 4 (DRD4) is polymorphic in many species, and several studies on birds (Fidler et al. 2007; Korsten et al. 2010; Garamszegi et al. 2014; Mueller et al. 2014; Riyahi et al. 2015; van Dongen et al. 2015; Holtmannn et al. 2016; but see Mueller et al. 2013; Edwards et al. 2015; Rollins et al. 2015), mice (Holmes et al. 2003), and rhesus macaques (Coyne et al. 2015) have indicated an association with exploration and boldness (Table 1) and in humans and mice even with lifespan, especially in females (Grady et al. 2013). Interestingly, both DRD4 and serotonin transporter (SERT) have pleiotropic effects also on sexual behaviors (Table 1). In humans and rats (*Rattus norvegicus*), DRD4 affects sexual desire, arousal, and function (Ben Zion et al. 2006; Melis et al. 2006). A recent study in dunnocks (*Prunella modularis*) shows how a number of single nucleotide polymorphisms (SNPs) in both DRD4 and SERT are sex-specifically associated with mating behavior (whether birds breed in monogamous pairs or in promiscuous groups), as well as risk taking (Holtmannn et al. 2016). Dunnocks have an unusually complicated mating system, combining social monogamy, polyandry, and polygyny, and the SNP effects have likely arisen in response to sex-specific selection on different reproductive strategies (Holtmann et al. 2016). However, fitness consequences of the alleles on each sex still need to be quantified. A gene *monoamine oxidase A* (MAOA) is another candidate gene in the dopaminergic and serotonin systems and associated with variation in aggression human and mouse males (Shih and Chen 1999; Raine 2008), as well as *nitric oxidase synthase 1* (NOS1) which has pleiotropic effects also on sexual function (see Table 1).

Parental care is an important component of POLS framework, in which caring for offspring is predicted to trade off with investment into fast reproduction and pace-of-life (Réale et al. 2010; Hämäläinen et al. 2018, topical collection on Pace-of-life syndromes). The neurobiology of parental care is sex-specific and involves neuropeptides, of which vasopressin (VP) and oxytocin (OXT) are the most potent ones (Bosch and Neumann 2012; Bales and Saltzman 2016; Dumais and Veenema 2016; Table 1). Both VP and OXT together with their receptors also affect sexual behaviors such as pair bonding and preference for monogamous pairing as well as mate guarding, social bonding and recognition, aggression, stress, and anxiety (Table 1, Fig. 1). VP-encoding gene is implicated in a recent study to underlie sex-specific differences in parental care between two closely related species of mice, oldfield mice (*Peromyscus polionotus*) and deer mice (*P. maniculatus*), that differ markedly in their mating system and level of offspring care (Bendesky et al. 2017). The species have diverged in 12 genomic regions associated with differences in parental behavior, most of which have sex-specific effects. This suggests that the paternal and maternal care differences between the species are governed by different mechanisms (Bendesky et al. 2017), which aligns well with the literature on neuronal regulatory pathways (Bosch and Neumann 2012; Bales and Saltzman 2016). Both VP and OXT are thus important candidate pathways that may govern not only within-species variation but also differences between species in behavioral syndromes associated with sex-specific divergence in mating systems and parental behavior.

### Mechanisms underlying pace-of-life

The mechanisms underlying evolutionary costs of reproduction form the basis of pace-of-life variation. Current reproduction may trade off with future reproductive effort or longevity, because resources are diverted from somatic maintenance, or because of the damage it inflicts. Studies on model organisms suggest that reproductive trade-offs arise from the links between resource acquisition, metabolism, reproduction, and lifespan. All of these components are commonly sexually dimorphic and thought to evolve due to sex-specific selection on resource allocation and sexual conflicts (e.g., effects of seminal fluid proteins, see below) (Adler and Bonduriansky 2014; Brooks and Garratt 2017). In line with roles of resource acquisition and allocation into longevity, dietary restriction (limiting calories, macronutrients or certain amino acids) extends lifespan and delays senescence in a range of organisms from yeasts to humans (Masoro 2005; Fontana et al. 2010; Le Couteur et al. 2016; Brooks and Garratt 2017). This effect is commonly sex-specific (Magwere et al. 2004; Tower 2006; Baar et al. 2016; Brooks and Garratt 2017), reflecting differences in dietary resource optimization between females and males (Maklakov et al. 2008; Brooks and Garratt 2017). Importantly, what matters for trade-offs is not simply variation in resource levels such as calories, but the balance of available nutrients: Reproductive rate and lifespan can be maximized by different diet components and these effects can be sex-specific (Brooks and Garratt 2017).

#### Nutrient sensing pathways

The genetic architecture governing the trade-off between current and future reproduction is poorly understood; however, the reproduction-longevity trade-offs has been investigated intensively, and frequently found to show sex differences (e.g., seed beetles: Fox et al. 2004; Bilde et al. 2009; Immonen et al. 2016a, *Drosophila*: Vieira et al. 2000; Burger and Promislow 2004). The most important pathways mediating reproductive costs, in terms of longevity, include those controlling growth and energy metabolism via nutrient sensing pathways, including insulin/insulin-like growth factor 1 (IGF1), the mechanistic target of rapamycin (mTOR), and sirtuins (Fontana et al. 2010; Brooks and Garratt 2017). IGF1 and growth hormone (GH) influence mammalian growth, and plasma levels of IGF1 correlate with the pace-of-life across species (Swanson and Dantzer 2014). Reducing the function of these growth-promoting signals leads to a greater elongation of lifespan in females than in males, as well as when mTOR activity is decreased, although the patterns also differ between species (Brooks and Garratt 2017).

Insulin-like growth factors (and homologs across species, e.g., CHICO in *Drosophila*, Clancy et al. 2001) and mTOR have important pleiotropic effects on other traits through which they not only regulate the reproduction-longevity trade-offs but also integration with sexually dimorphic growth rate and development time, as well as secondary sexual signaling and attractiveness via cuticular hydrocarbon signaling (Tatar et al. 2003; Emlen et al. 2012). While these genes have not been studied in the POLS context, they may also directly influence behaviors, possibly through involvement in neuroendocrine control. For example, rapamycin, which blocks some of the mTOR functions, is implicated in (pathological) behaviors of mice (Cleary et al. 2008; Halloran et al. 2012), and neuroendocrine receptors of insulin-producing cells in *Drosophila* brain affect aggressive and courtship behaviors (Luo et al. 2014). An association of insulin signaling with juvenile hormone secretion has also been implicated as a mechanism behind behavioral variation in *Drosophila* (Belgacem and Martin 2006), thus linking the expression of behavior, life history, and physiology.

#### Glucocorticoids

Glucocorticoids (GC) are an important candidate mediator of POLS because they orchestrate energy allocation and storage and allow individuals to respond to the environment adaptively to maximize fitness through their influence on investment into reproduction, immunity, and resource acquisition (Sapolsky et al. 2000; Bonier et al. 2009). Glucocorticoids are responsible for quick mobilization of energy reserves and suppression of non-essential metabolic processes. Therefore, high GC levels can be an adaptive response to acute stressors but detrimental over time (Sapolsky 2005). GCs have also been linked to “coping styles” under stress (proactive and reactive) that potentially link life history, physiology, and behavioral traits in predictable ways (Øverli et al. 2006; Carere et al. 2010; Koolhaas et al. 2010; Silva et al. 2010; Tudorache et al. 2013). A proactive stress-coping involves using aggression to counteract the stressful stimulus, while reactive coping style is characterized by immobility and avoidance of aggression, associated with differences in hypothalamus–pituitary–adrenal (HPA) axis (Table 2) responsiveness and brain monoamine neurochemistry (Øverli et al. 2007). The sexes commonly differ in their behavioral and physiological response to stress (e.g., Bale and Epperson 2015) as well as in their immune response, because glucocorticoids interact with sex hormones (Handa et al. 1994; Silva 1999; Lighthall et al. 2009; Bourke et al. 2012). Although the patterns are not yet clear, glucocorticoid baseline or stress-induced levels tend to be higher in females (Kudielka and Kirschbaum 2005; Hämäläinen et al. 2015). It is plausible that sex-specific effects of the environment may be a result of sex-specific responsiveness to stressors (Killen et al. 2013).

#### Metabolic genes

The POLS framework hypothesizes a link between energy metabolism, behavioral, and life history traits due to the simple reason that limited amounts of energy are needed to fuel all competing processes of the body. Individuals and species vary in how much energy is available to them and how efficiently it may be converted to different processes. Because of this, a correlation between metabolic rate and pace-of-life is expected (Careau et al. 2008, 2010; Biro and Stamps, 2010; Careau and Garland 2012; Glazier 2014). Also, females and males often differ in their metabolic requirements (e.g., Rogowitz and Chappell 2000), and the mechanisms that govern the energy balance should therefore evolve under sex-specific selection. In line with this (mass-specific), metabolic rate commonly differs between the sexes (e.g., Kolluru et al. 2004; Krasnov et al. 2004; Berger et al. 2014a, b, 2016; Kurbalija Novicic et al. 2014; Rønning et al. 2014; but see Krams et al. 2017), and a recent study on a seed beetle (*Acanthoscelides obtectus*) shows how divergence in the pace-of-life is associated with metabolic rate in a sex-specific way (Arnqvist et al. 2017).

The relationship between metabolic rate and other POLS traits suggests involvement of metabolic genes with major pleiotropic effects that may differ in the sexes (Stamps 2007; Careau et al. 2008; Biro and Stamps 2010). Although the genetic architecture of metabolic rate is not yet fully understood, it involves epistatic interactions between gene products from both mitochondrial (mtDNA) and nuclear genomes (Arnqvist et al. 2010; Immonen et al. 2016b) that together form the three main energy pathways generating ATP (glycolysis, tricarboxylic acid cycle and oxidative phosphorylation). This kind of mitonuclear epistasis not only influences life history traits but importantly also their covariance with systematic behaviors in a seed beetle *C. maculatus*, consistent with POLS (Lovlie et al. 2014). In *A. obtectus* beetles, different mtDNA haplotypes are associated with “slow” and “fast” pace-of-life and sex-specific mitochondrial bioenergetics (Dordevic et al. 2017). Indeed, the effects of mtDNA variation and mitonuclear epistasis are often sex-specific (Dobler et al. 2014; Immonen et al. 2016a). This is predicted because of the maternal inheritance of mitochondria, which allows mtDNA to respond to selection only through females, and therefore the potential for accumulation of male-harming mtDNA mutations (i.e., “mother’s curse,” Gemmell et al. 2004; Innocenti et al. 2011). As a result, males can show more phenotypic variation arising from mtDNA variation than females, as demonstrated for aging and fertility in *D. melanogaster* (Camus et al. 2012). Selection on males to restore and improve male-specific mitochondrial function can operate via nuclear genes (e.g., Gallach and Betran 2011), which should lead to a genetic architecture of metabolism that involves partly different genes and regulatory patterns in males and females. In a bank vole *M. glareolus*, mtDNA haplotype variation influences the correlation between metabolic rate and proactive behavior depending on the nuclear genetic background, but only in males (Sichova et al. 2014). In accordance with theory, these patterns suggest that mitonuclear cross talk has the power to generate sex-specific patterns in POLS trait correlations that may also be sub-optimal in males due to the sex difference in the efficacy of selection. More work is however needed to understand the mechanistic relationship between the metabolic pathways and “personality” behaviors (see also Krams et al. 2017).

#### Male seminal fluid proteins affect female behavior and life history trade-offs

An interesting but less often considered mechanism affecting exclusively female reproductive investment and trade-offs with lifespan includes molecular interactions that occur upon mating with males. Due to the different evolutionary interests of the sexes over reproductive rates (Parker 1979; Arnqvist and Rowe 2005), males can be selected to manipulate female reproductive effort towards current reproductive event via transferring a cocktail of seminal fluid proteins and peptides (SFPs) during mating (Poiani 2006). One such molecule, the sex peptide (SP) of *D. melanogaster*, influences female fecundity, re-mating rate, feeding, activity patterns, and aggression, as well as immunity activation (Avila et al. 2011; Sirot et al. 2015; Bath et al. 2016). SP improves male fitness (Fricke et al. 2010), at the expense of female lifespan (Chapman et al. 1995). Such effects can arise at least partly through trade-offs: males induce fecundity in females, who pay an extra latent cost of reproduction through accelerated aging. However, SFPs reduce lifespan of even sterilized females in *D. melanogaster* (Barnes et al. 2008; see Maures et al. 2014 for similar findings in *Caenorhabditis elegans*). The mechanisms behind these effects in female *Drosophila* are not yet known, but hormonal control via juvenile hormone may play a role (Moshitzky et al. 1996; Yamamoto et al. 2013). The fact that molecular interactions can have such profound consequences on key life history and behavioral traits in females implies that many traits in female POLS may be subject to both direct genetic effects of the females and indirect genetic effects that depend on male genotype (Immonen et al. 2016a). What consequences such a complex genetic control has for the evolution of POLS is an important question for elucidating the evolutionary trajectories in each sex.

#### Sex steroids and juvenile hormone are master regulators of sex-specific POLS

As discussed earlier, the sex hormones estrogen and testosterone are essential for sexually dimorphic behaviors in vertebrates. Estrogen is responsible for generating the repertoire of sexual and territorial behaviors in both sexes, while testosterone modifies the intensity of these behaviors (Ogawa et al. 2000; Kudwa and Rissman 2003; Kudwa et al. 2006; Raskin et al. 2009; Wu et al. 2009; Juntti et al. 2010; Wu and Shah 2011). Sex hormones are important mediators of sexual dimorphism in systematic behaviors, such as those forming animal “personality,” because they influence not only the development and modification of sex-specific behaviors in adults but also behaviors such as aggression and anxiety (Book et al. 2001). The relative roles for testosterone and estrogen in aggression differ in females and males (Hau 2007), which can allow decoupling the effects in the sexes (Cain et al. 2016). Similar sex specificity underlies anxiety: Studies in rodents discovered that a particular isoform of the estrogen receptor (ERβ) decreases anxiety in females (Imwalle et al. 2005; Lund et al. 2005) while increased levels of circulating testosterone decrease anxiety in both sexes (Celec et al. 2015) via androgen receptors (Zuloaga et al. 2008). Indeed, both estrogen receptors α and β regulate oxytocin levels, through which they influence territorial aggression, anxiety, parental behaviors, and mate preference (Table 1). Genetic variation in sex hormone receptors such as ERα and ERβ is therefore one putative way to achieve sex-specific functional variation. In the white-throated sparrow (*Zonotrichia albicollis*), plumage coloration and sexually dimorphic polymorphism in reproductive strategies is determined by alternative alleles at an inversion-based “supergene” including ERα (Tuttle et al. 2016). The behavioral differences in territorial aggression and parental care between males of the different morphs are mediated by expression differences in ERα, while behavioral differences in female sparrows have a different neural mechanism than in males (Horton et al. 2014). ERα causes similar behavioral differences also in prairie voles: Males showing more promiscuity and less parental care express ERα at a higher level in the same brain region as the white-morph sparrows (Cushing et al. 2008).

Testosterone has received particular interest in evolutionary biology because of its role in mediating trade-offs between reproductive allocation and self-preservation via physiological effects such as lower investment in immune defense (Muehlenbein and Bribiescas 2005) and between mating and parental effort (McGlothlin et al. 2007). For example, increased aggressiveness may benefit a male in terms of acquisition and defense of resources or mates, but also influence parasite loads and immunocompetence (Zuk and Stoehr 2002; Muehlenbein and Bribiescas 2005; Hayward 2013) as well as the expression of secondary sexual characters (Roberts et al. 2004). Testosterone has also a negative/reducing effect on longevity, either directly or through its effects on behavior, while estrogen levels extend lifespan (Brooks and Garratt 2017). Testosterone also plays an important role in limiting expression of alternative male phenotypes (i.e., polymorphism in physiological, behavioral, morphological and life history differences) to males (Hau 2007). The ruff (*Philomachus pugnax)* is a good example of such a polymorphic reproductive strategy: Males exhibit three alternative mating tactics where they differ in aggression, hormone levels, mating behaviors, and morphology (Lank et al. 1995, 2013; Kupper et al. 2016). Females can also express morph differences if their testosterone levels are artificially increased (Lank et al. 1999).

Although sex steroids play a pivotal part in controlling sex-biased expression, thereby decreasing the genetic correlation between the sexes (Cox et al. 2017), they can also be a source of sexual conflict (Mokkonen et al. 2011; Mills et al. 2012). Sex-specific and sexually antagonistic selection may help to explain some of the surprising differences observed between species (Hau and Goymann 2015) and offer one mechanism for how commonly observed variation in testosterone levels among individuals (Williams 2008) can be maintained (Hämäläinen et al. 2018, topical collection on Pace-of-life syndromes). To improve our understanding of how sex hormones, and particularly testosterone effects on behavior, and life history trade-offs evolve, we need to start to factor in their fitness impacts on each sex and to estimate the genetic architecture of the trait correlations within and across sexes. We suspect that multitrait phenotypes involving sex steroids may commonly have sex-specific genetic architecture, as implicated in masked boobies (*Sula dactylatra*) (Fargallo et al. 2014).

Juvenile hormone has been raised as a candidate testosterone-analogue responsible for mediating reproductive and immune investment in invertebrates (Rantala et al. 2003) and has potential to influence polymorphisms and polyphenisms (Nijhout and Wheeler 1982). It has effects on multiple life history components including, for instance, development (Schal et al. 1997), metamorphosis (Flatt et al. 2005), sexual maturation (Schal et al. 1997), (sexual) behavior (Strambi et al. 1997; Flatt et al. 2005), reproduction, diapause, stress resistance, aging (Flatt et al. 2005), and gene expression (reviewed in Jones 1995; Wyatt and Davey 1996). Juvenile hormone also plays a role in sex-specific signaling by mediating sexual horn dimorphism in horned beetles (Shelby et al. 2007) and possibly in sexual size dimorphism (Stillwell et al. 2010). The hormone is broadly involved in parental care, with levels during parental care differing in the sexes (Trumbo 2002; Panaitof et al. 2004). Although early evidence suggested that male juvenile hormone titres may be higher than those of females (Gilbert and Schneiderman 1961), very few studies have measured juvenile hormone levels or its effects in both sexes (Stillwell et al. 2010). Thus, broader sex differences in its activity remain to be resolved.

#### Sexual dimorphism in body size and integration with POLS

Sexual size dimorphism is widespread and may be directly associated with sex differences in life history, behavioral, and physiological traits through differing resource acquisition-allocation patterns, sex-specific effects of the environment, and sexual selection (Shine 1989; Magurran and Garcia 2000; Isaac 2005; Stillwell et al. 2010). Body size has traditionally been considered a part of the fast-slow continuum, where small adult body size was thought to covary with a fast life history and large body size with a slow one (Reynolds 2003). This categorization is not, however, straightforward and is further complicated by taxon-specific sexual size dimorphism (Abouheif and Fairbairn 1997; Fairbairn 1997).

Nevertheless, body size is an important variable that can influence suites of traits simultaneously, and subsequently sex-specific fitness through these associations (see, e.g., Berg and Maklakov 2012; Berger et al. 2014a, b; Gaillard et al. 2016). For example, body size scales with the size of the digestive tract, basal metabolic rate, and to some extent feeding behavior (Perez-Barberia et al. 2008). When comparing species, allometric scaling of morphology plays an important role in evolutionary trajectories (Gould 1966), and therefore, it is likely that body size also influences the evolution of POLS. Sex-specific integration of body size and POLS may be expected for example when size dimorphism is related to sex differences in resource requirements, predation, or pathogen infection risk (Peters 1983), which can cause sex-specific selection on foraging activity, boldness, or behavioral activity and immunity, respectively. Body size may therefore be a central player underlying sex-specific genetic correlations among POLS traits, for example through shared molecular mechanisms influencing growth and variation in pace-of-life. For example *VGLL3* and insulin/IGF1 signaling pathway both affect body size and development time with sex-specific effects, with the latter also important for longevity trade-offs (see above) (Table 2, Fig. 1). Melanocortin system is another interesting pathway, affecting not only body size via adipose tissue regulation, immunity, and stress but also systematic variation in aggressive and sexual behaviors (Table 1, Fig. 1).

The degree of integration (i.e., strength of covariation, Klingenberg 2008) with body size will affect the potential for independent evolution of the specific POLS traits, with strong integration constraining independent evolution of the traits, thus maintaining the syndrome. However, the level of integration between body size and different POLS trait categories differs, being greater with physiology, such as basal metabolic rate (Konarzewski and Książek 2012), and lower with life history traits, such as developmental time (e.g., Blanckenhorn et al. 2007). The integration of body size and POLS traits also varies between species, and particularly so in those with high sexual dimorphism (Fairbairn and Preziosi 1994; Fairbairn 1997). If the variation in integration with different POLS traits varies also sex-specifically, there is opportunity for independent trait evolution and sex-specific covariances between POLS traits. Given that body size is a common target of sex-specific selection (Fairbairn et al. 2007), more work should be devoted in understanding coevolution of body size and POLS in the sexes coupled with identifying the molecular underpinnings of the genetic integration.

## Conclusions and future prospects

The POLS framework stems from the observation that animals vary consistently in their behavior, which could both arise from, as well as be the source of, variation in life history traits along the pace-of-life continuum, integrated through physiology. Although behavioral differences between the sexes are highly prevalent (e.g., Schuett et al. 2010) and their study has been central to behavioral ecology from the start of this field, studies examining the evolutionary causes and mechanisms underlying sex differences in POLS have only now started to appear. On the other hand, sex differences have been a focus of much research in life history and physiological contexts, and particularly in animals that can be reared in the laboratory. Our aim in this article was to bridge some of this gap by bringing together key concepts and insights from the fields of behavior, life history, and reproductive genetics, to inspire future work to look more closely into how genetic covariances underlying POLS are generated in each sex.

In particular, we emphasize the need for (quantitative) genetic studies on covariation between life history trade-offs between current and future reproduction and behavioral syndromes in each sex, which are currently rare. This entails studying correlations between traits in more than two POLS categories at a time (e.g., immunity and behavior), which is currently the dominating approach. Also, given that the POLS composition is expected to vary between the sexes and species depending, e.g., on the mating system and environment (Hämäläinen et al. 2018, topical collection on Pace-of-life syndromes), care should be taken to study the most relevant trait combinations for a given study system. Comparative studies across species with different mating systems would be particularly useful in testing the consequence of variation in sex-specific selection on the genetic architecture (i.e., G- and B-matrices) of POLS in the sexes. Experimental evolution and artificial selection studies can be used to study these at the micro-evolutionary scale.

Molecular genetics and pharmacological studies suggest that shared pathways underlie many POLS traits, opening opportunities to study genetic variation in these pathways and the relative roles of gene pleiotropy and linkage in forming genetic correlations. Although pleiotropy is commonly seen as a constraint of independent trait evolution, POLS framework can offer a fruitful context in which to study whether selection may have favored genetic coupling of traits under shared pathways. Studies of sex-specific genetic and hormonal effects on POLS traits have started to emerge and suggest that sex specificity is common in their effects on both behavioral and life history traits. What we need to understand next is what consequences such effects have on trait correlations and what are their fitness impacts in each sex, and thus consequences on the evolutionary dynamics of POLS. Failure to incorporate the putative sex specificity and the integrated nature of traits in the studies of POLS can risk overlooking important mechanisms that can only be found in one sex.

Studying POLS in the context of sex differences forces us also to consider how primary and secondary sexual characteristics covary with POLS traits. For example, sex-specific selection on melanin pigmentation—due to its role as a sexual signal or in immunity—may affect POLS due to the pleiotropic effects of melanocortins. Sexually dimorphic body size and its role in both reproductive trade-offs and behavioral syndromes is another example. Although predictions for such traits are not straightforward in the POLS framework (e.g., Réale et al. 2010), incorporating sexual traits would improve our understanding of how selection operates on integrated phenotypes as a whole.

It is interesting to note that observing sex differences at the molecular level may not translate into sex differences at the phenotypic level: Some sex differences in gene action may have evolved to enable the sexes to display similar phenotypes, such as behaviors (De Vries 2004). For example, evolution of monogamy and convergent reproductive roles from a polygamous ancestral state may require further sexually dimorphic or sex-limited changes because of the pre-existing sex differences. This would suggest that—depending on the evolutionary history—the genetic architecture of POLS may be sex-specific even when the phenotype is monomorphic in the sexes. Comparative studies between species that differ in reproductive roles, and thus in selection for sexual dimorphism, may be particularly useful for illuminating the extent to which variation in the sexual dimorphism in POLS is reflected on dimorphism in the underlying genetic architecture.

In this article, we have mainly focused on discussing how sex differences within species arise and what consequences these may have for POLS at a population level. Given that differences in reproductive trade-offs between the sexes as well as species can create a similar gradient of POLS, one important question is whether there is a conserved genetic basis underlying this gradient for both individuals of the different sexes as well as species. Many of the genes and pathways discussed here are largely conserved (e.g., nutrient sensing pathways, melanocortins, DRD4, SERT; Tables 1 and 2) opening up this opportunity, and comparative work would help to test whether “fast” and “slow” strategies indeed share a genetic basis across sexes and species.

Currently, there is a strong bias in the molecular studies in favor of model organisms. Several molecular and quantitative genetic tools are now, however, also available for studying personality and life history variation in non-model organisms, allowing answering questions such as what genetic variants are segregating in natural populations and how the genetic effects may interact with the natural environment. The study on New Zealand population of dunnocks is an example of utilizing a human candidate gene approach to unravel the genetic basis of personality and mating behavior variation in the wild (Holtmann et al. 2016). Sequencing candidate genes is an inexpensive alternative to looking for new ones with quantitative trait loci (QTL) mapping or using a genome wide association study (GWAS) technique that utilizes genome-wide information of single nucleotide polymorphisms (SNPs). However, with the candidate gene approach, care needs to be taken to properly account for population structure by using several neutral genetic markers. Discovering novel candidate genes also in non-model organisms is now made possible by the rapid rise of affordable sequencing technologies, such as RNA-seq and RAD-seq, that can also be used with species without previous genetic tools (da Fonseca et al. 2016). RNA-seq can also be used to study the role of sex-biased gene expression in regulating sexual dimorphism in POLS. Functional studies of genes to elucidate putative sex-specific effects are essential but have only recently started to emerge (e.g., Dumais and Veenema 2016). These are now made available also in non-model organisms with CRISPR/Cas9 technique (Singh et al. 2015).

We hope that this review will encourage more endeavors of studying the mechanistic basis of POLS but also spark general interest in appreciating what makes males and females unique and how far-reaching consequences these differences may have on the evolution of POLS.

## Electronic supplementary material

ESM 1 (DOCX .5.5 kb)

Fig. S1 (DOCX 476 kb)

## References

[CR1] Abouheif E, Fairbairn DJ (1997). A comparative analysis of allometry for sexual size dimorphism: assessing Rensch’s rule. Am Nat.

[CR2] Adler MI, Bonduriansky R (2014). Sexual conflict, life span, and aging. CSH Perspect Biol.

[CR3] Albert AYK, Otto SP (2005). Sexual selection can resolve sex-linked sexual antagonism. Science.

[CR4] Alicchio R, Palenzona DL (1971). Changes of sexual dimorphism values in *Drosophila melanogaster*. Boll Zool.

[CR5] Andersson MB (1994). Sexual selection.

[CR6] Armbruster WS, Pelabon C, Bolstad GH, Hansen TF (2014). Integrated phenotypes: understanding trait covariation in plants and animals. Philos T Roy Soc B.

[CR7] Arnold SJ (1992). Constraints on phenotypic evolution. Am Nat.

[CR8] Arnold AP (2009). The organizational-activational hypothesis as the foundation for a unified theory of sexual differentiation of all mammalian tissues. Horm Behav.

[CR9] Arnold AP, Xu J, Grishman W, Chen X, Kin YH, Itoh Y (2004). Minireview: sex chromosomes and brain sexual differentiation. Endocrinology.

[CR10] Arnqvist G, Rowe L (2005). Sexual Conflict.

[CR11] Arnqvist G, Tuda M (2010). Sexual conflict and the gender load: correlated evolution between population fitness and sexual dimorphism in seed beetles. Proc R Soc Lond B.

[CR12] Arnqvist G, Dowling DK, Eady P, Gay L, Tregenza T, Tuda M, Hosken DJ (2010). Genetic architecture of metabolic rate: environment specific epistasis between mitochondrial and nuclear genes in an insect. Evolution.

[CR13] Arnqvist G, Stojkovic B, Liljestrand-Rönn J, Immonen E (2017). The pace-of-life: a sex-specific link between metabolic rate and life history in bean beetles. Funct Ecol.

[CR14] Avale ME, Falzone TL, Gelman DM, Low MJ, Grandy DK, Rubinstein M (2004). The dopamine D4 receptor is essential for hyperactivity and impaired behavioral inhibition in a mouse model of attention deficit/hyperactivity disorder. Mol Psychiatry..

[CR15] Avila FW, Sirot LK, LaFlamme BA, Rubinstein CD, Wolfner MF (2011). Insect seminal fluid proteins: identification and function. Annu Rev Entomol.

[CR16] Baar EL, Carbajal KA, Ong IM, Lamming DW (2016). Sex- and tissue-specific changes in mTOR signaling with age in C57BL/6J mice. Aging Cell.

[CR17] Bale TL, Epperson CN (2015). Sex differences and stress across the lifespan. Nat Neurosci.

[CR18] Bales KL, Saltzman W (2016). Fathering in rodents: Neurobiological substrates and consequences for offspring. Horm Behav..

[CR19] Barker BS, Phillips PC, Arnold SJ (2010). A test of the conjecture that G-matrices are more stable than B-matrices. Evolution.

[CR20] Barnes AI, Wigby S, Boone JM, Partridge L, Chapman T (2008). Feeding, fecundity and lifespan in female *Drosophila melanogaster*. Proc R Soc Lond B.

[CR21] Barrett CE, Modi ME, Zhang BC, Walum H, Inoue K, Young LJ (2014). Neonatal melanocortin receptor agonist treatment reduces play fighting and promotes adult attachment in prairie voles in a sex-dependent manner. Neuropharmacology..

[CR22] Barson NJ, Aykanat T, Hindar K (2015). Sex-dependent dominance at a single locus maintains variation in age at maturity in salmon. Nature.

[CR23] Bateman AJ (1948). Intra-sexual selection in *Drosophila*. Heredity.

[CR24] Bath E, Bowden S, Peters C, Reddy A, Tobias JA, Easton-Calabria E, Seddon N, Goodwin SF, Wigby S (2016). Sperm and sex peptide stimulate aggression in female *Drosophila*. Nat Ecol Evol.

[CR25] Belgacem YH, Martin JR (2006). Disruption of insulin pathways alters trehalose level and abolishes sexual dimorphism in locomotor activity in *Drosophila*. J Neurobiol.

[CR26] Ben Zion IZ, Tessler R, Cohen L (2006). Polymorphisms in the *dopamine D4 receptor gene* (*DRD4*) contribute to individual differences in human sexual behavior: desire, arousal and sexual function. Mol Psychiatry.

[CR27] Bendesky A, Kwon YM, Lassance JM, Lewarch CL, Yao SQ, Peterson BK, He MX, Dulac C, Hoekstra HE (2017). The genetic basis of parental care evolution in monogamous mice. Nature.

[CR28] Berg EC, Maklakov AA (2012). Sexes suffer from suboptimal lifespan because of genetic conflict in a seed beetle. Proc R Soc Lond B.

[CR29] Berger D, Grieshop K, Lind MI, Goenaga J, Maklakov AA, Arnqvist G (2014). Intralocus sexual conflict and environmental stress. Evolution.

[CR30] Berger D, Berg EC, Widegren W, Arnqvist G, Maklakov AA (2014). Multivariate intralocus sexual conflict in seed beetles. Evolution.

[CR31] Berger D, Martinossi I, Grieshop K, Lind MI, Maklakov A, Arnqvist G (2016). Intralocus sexual conflict and the tragedy of the commons in seed beetles. Am Nat.

[CR32] Bilde T, Maklakov AA, Meisner K, la Guardia L, Friberg U (2009). Sex differences in the genetic architecture of lifespan in a seed beetle: extreme inbreeding extends male lifespan. BMC Evol Biol.

[CR33] Bird MA, Schaffer HE (1972). Study of genetic basis of sexual dimorphism for wing length in *Drosophila melanogaster*. Genetics.

[CR34] Biro PA, Stamps JA (2010). Do consistent individual differences in metabolic rate promote consistent individual differences in behavior?. Trends Ecol Evol.

[CR35] Björklund M, Husby A, Gustafsson L (2013). Rapid and unpredictable changes of the G-matrix in a natural bird population over 25 years. J Evol Biol.

[CR36] Blanckenhorn WU, Dixon AFG, Fairbairn DJ (2007). Proximate causes of Rensch’s rule: does sexual size dimorphism in arthropods result from sex differences in development time?. Am Nat.

[CR37] Bonduriansky R, Chenoweth SF (2009). Intralocus sexual conflict. Trends Ecol Evol.

[CR38] Bonduriansky R, Maklakov A, Zajitschek F, Brooks R (2008). Sexual selection, sexual conflict and the evolution of ageing and life span. Funct Ecol.

[CR39] Bonier F, Martin PR, Moore IT, Wingfield JC (2009). Do baseline glucocorticoids predict fitness?. Trends Ecol Evol.

[CR40] Book AS, Starzyk KB, Quinsey VL (2001). The relationship between testosterone and aggression: a meta-analysis. Aggress Violent Behav.

[CR41] Bosch OJ, Neumann ID (2012) Both oxytocin and vasopressin are mediators of maternal care and aggression in rodents: from central release to sites of action. Horm Behav. 61(3):293–303. 10.1016/j.yhbeh.2011.11.002 10.1016/j.yhbeh.2011.11.002 22100184

[CR42] Bourke CH, Harrell CS, Neigh GN (2012). Stress-induced sex differences: adaptations mediated by the glucocorticoid receptor. Horm Behav.

[CR43] Brodie ED (1989). Genetic correlations between morphology and antipredator behavior in natural-populations of the garter snake *Thamnophis ordinoides*. Nature.

[CR44] Brodie ED (1992). Correlational selection for color pattern and antipredator behavior in the garter snake *Thamnophis ordinoides*. Evolution.

[CR45] Brooks RC, Garratt MG (2017). Life history evolution, reproduction, and the origins of sex-dependent aging and longevity. Ann N Y Acad Sci.

[CR46] Burger JM, Promislow DE (2004). Sex-specific effects of interventions that extend fly life span. Sci Aging Knowl Environ.

[CR47] Cain K, Jawor JM, McGlothlin JW, Ketterson ED, Atwell JW (2016). Individual variation and selection on hormone-mediated phenotypes in male and female dark-eyed juncos. Snowbird: integrative biology and evolutionary diversity in the junco.

[CR48] Camus MF, Clancy DJ, Dowling DK (2012). Mitochondria, maternal inheritance, and male aging. Curr Biol.

[CR49] Camus MF, Wolf JB, Morrow EH, Dowling DK (2015). Single nucleotides in the mtDNA sequence modify mitochondrial molecular function and are associated with sex-specific effects on fertility and aging. Curr Biol..

[CR50] Careau V, Garland T (2012). Performance, personality, and energetics: correlation, causation, and mechanism. Physiol Biochem Zool.

[CR51] Careau V, Thomas D, Humphries MM, Réale D (2008). Energy metabolism and animal personality. Oikos.

[CR52] Careau V, Réale D, Humphries MM, Thomas DW (2010). The pace-of-life under artificial selection: personality, energy expenditure, and longevity are correlated in domestic dogs. Am Nat.

[CR53] Carere C, Caramaschi D, Fawcett TW (2010). Covariation between personalities and individual differences in coping with stress: converging evidence and hypotheses. Curr Zool.

[CR54] Celec P, Ostatníková D, Hodosy J (2015). On the effects of testosterone on brain behavioral functions. Front Neurosci.

[CR55] Chapman T, Liddle LF, Kalb JM, Wolfner MF, Partridge L (1995). Cost of mating in *Drosophila melanogaster* females is mediated by male accessory-gland products. Nature.

[CR56] Cheng C, Kirkpatrick M (2016). Sex-specific selection and sex-biased gene expression in humans and flies. PLoS Genet.

[CR57] Cheverud JM (1984). Quantitative genetics and developmental constraints on evolution by selection. J Theor Biol.

[CR58] Clancy DJ, Gems D, Harshman LG, Oldham S, Stocker H, Hafen E, Leevers SJ, Partridge L (2001). Extension of life-span by loss of CHICO, a *Drosophila* insulin receptor substrate protein. Science.

[CR59] Cleary C, Linde JAS, Hiscock KM, Hadas I, Belmaker RH, Agam G, Flaisher-Grinberg S, Einat H (2008). Antidepressive-like effects of rapamycin in animal models: implications for mTOR inhibition as a new target for treatment of affective disorders. Brain Res Bull.

[CR60] Cone RD (2005). Anatomy and regulation of the central melanocortin system. Nat Neurosci.

[CR61] Connallon T, Clark AG (2010). Sex-linkage, sex-specific selection, and the role of recombination in the evolution of sexually dimorphic gene expression. Evolution.

[CR62] Connallon T, Clark AG (2011). The resolution of sexual antagonism by gene duplication. Genetics.

[CR63] Connallon T, Clark AG (2014). Evolutionary inevitability of sexual antagonism. Proc R Soc B.

[CR64] Cox RM, Calsbeek R (2009). Sexually antagonistic selection, sexual dimorphism, and the resolution of intralocus sexual conflict. Am Nat.

[CR65] Cox RM, Cox CL, McGlothlin JW, Card DC, Andrew AL, Castoe TA (2017). Hormonally mediated increases in sex-biased gene expression accompany the breakdown of between-sex genetic correlations in a sexually dimorphic lizard. Am Nat.

[CR66] Coyne SP, Lindell SG, Clemente J, Barr CS, Parker KJ, Maestripieri D (2015). *Dopamine D4* receptor genotype variation in free-ranging rhesus macaques and its association with juvenile behavior. Behav Brain Res.

[CR67] Cushing BS, Perry A, Musatov S, Ogawa S, Papademetriou E (2008). Estrogen receptors in the medial amygdala inhibit the expression of male prosocial behavior. J Neurosci.

[CR68] da Fonseca RR, Albrechtsen A, Themudo GE, Ramos-Madrigal J, Sibbesen JA, Maretty L, Zepeda-Mendoza ML, Campos PF, Heller R, Pereira RJ (2016). Next-generation biology: sequencing and data analysis approaches for non-model organisms. Mar Genom.

[CR69] Dall SRX, Houston AI, McNamara JM (2004). The behavioural ecology of personality: consistent individual differences from an adaptive perspective. Ecol Lett.

[CR70] Delph LD, Steven JC, Anderson IA, Herlihy CR, Brodie ED (2011). Elimination Of a genetic correlation between the sexes via artificial correlational selection. Evolution.

[CR71] De Vries GJ (2004). Minireview: sex differences in adult and developing brains: compensation, compensation, compensation. Endocrinology.

[CR72] De Vries GJ, Rissman EF, Simerly RB, Yang LY, Scordalakes EM, Auger CJ, Swain A, Lovell-Badge R, Burgoyne PS, Arnold AP (2002). A model system for study of sex chromosome effects on sexually dimorphic neural and behavioral. J Neurosci.

[CR73] Dean R, Mank JE (2014). The role of sex chromosomes in sexual dimorphism: discordance between molecular and phenotypic data. J Evol Biol.

[CR74] Delahaie B, Charmantier A, Chantepie S, Garant D, Porlier M, Teplitsky C (2017) Conserved G-matrices of morphological and life-history traits among continental and island blue tit populations. Heredity. 119: 76–87. 10.1038/hdy.2017.15) 10.1038/hdy.2017.15 PMC5510724 28402327

[CR75] Delph LD, Steven JC, Anderson IA, Herlihy CR, Brodie III ED. 2011 Elimination Of a genetic correlation between the sexes via artificial correlational selection. Evolution 65(10):2872–2880 10.1111/j.1558-5646.2011.01350.x 21967428

[CR76] Dobler R, Rogell B, Budar F, Dowling DK (2014). A meta-analysis of the strength and nature of cytoplasmic genetic effects. J Evol Biol.

[CR77] Dochtermann NA (2011). Testing Cheverud’s conjecture for behavioral correlations and behavioral syndromes. Evolution.

[CR78] Dochtermann NA, Schwab T, Sih A (2015). The contribution of additive genetic variation to personality variation: heritability of personality. Proc R Soc B.

[CR79] Dordevic M, Stojkovic B, Savkovic U, Immonen E, Tucic N, Lazarevic J, Arnqvist G (2017). Sex-specific mitonuclear epistasis and the evolution of mitochondrial bioenergetics, ageing, and life history in seed beetles. Evolution.

[CR80] Ducrest AL, Keller L, Roulin A (2008). Pleiotropy in the melanocortin system, coloration and behavioural syndromes. Trends Ecol Evol.

[CR81] Dumais KM, Veenema AH (2016). Vasopressin and oxytocin receptor systems in the brain: sex differences and sex-specific regulation of social behavior. Front Neuroendocrinol.

[CR82] Edwards HA, Hajduk GK, Durieux G, Burke T, Dugdale HL (2015). No association between personality and candidate gene polymorphisms in a wild bird population. PLoS One.

[CR83] Ellegren H, Parsch J (2007). The evolution of sex-biased genes and sex-biased gene expression. Nat Rev Genet.

[CR84] Emaresi G, Bize P, Altwegg R, Henry I, van den Brink V, Gasparini J, Roulin A (2014). Melanin-specific life-history strategies. Am Nat.

[CR85] Emlen DJ, Warren IA, Johns A, Dworkin I, Lavine LC (2012). A mechanism of extreme growth and reliable signaling in sexually selected ornaments and weapons. Science.

[CR86] Fairbairn DJ (1997). Allometry for sexual size dimorphism: pattern and process in the coevolution of body size in males and females. Annu Rev Ecol Syst.

[CR87] Fairbairn DJ, Preziosi RF (1994). Sexual selection and the evolution of allometry for sexual size dimorphism in the water strider, *Aquarius remigis*. Am Nat.

[CR88] Fairbairn DJ, Blanckenhorn WJ, Székely T (2007). Sex, size and gender roles: evolutionary studies of sexual size dimorphism.

[CR89] Faraone SV, Doyle AE, Mick E, Biederman J (2001). Meta-analysis of the association between the 7-repeat allele of the dopamine D(4) receptor gene and attention deficit hyperactivity disorder. Am J Psychiatry.

[CR90] Fargallo JA, Velando A, Lopez-Rull I, Ganan N, Lifshitz N, Wakamatsu K, Torres R (2014). Sex-specific phenotypic integration: endocrine profiles, coloration, and behavior in fledgling boobies. Behav Ecol.

[CR91] Fidler AE, van Oers K, Drent PJ, Kuhn S, Mueller JC, Kempenaers B (2007). *DRD4* gene polymorphisms are associated with personality variation in a passerine bird. Proc R Soc Lond B.

[CR92] Flatt T, Tu MP, Tatar M (2005). Hormonal pleiotropy and the juvenile hormone regulation of *Drosophila* development and life history. BioEssays.

[CR93] Fontana L, Partridge L, Longo VD (2010). Extending healthy life span-from yeast to humans. Science.

[CR94] Fox CW, Czesak ME, Wallin WG (2004). Complex genetic architecture of population differences in adult lifespan of a beetle: nonadditive inheritance, gender differences, body size and a large maternal effect. J Evol Biol.

[CR95] Fricke C, Martin OY, Bretman A, Bussiere LF, Chapman T (2010). Sperm competitive ability and indices of lifetime reproductive success. Evolution.

[CR96] Fry JD (2010). The genomic location of sexually antagonistic variation: some cautionary comments. Evolution.

[CR97] Futuyma DJ (2010). Evolutionary constraint and ecological consequences. Evolution.

[CR98] Gaillard J-M, Lemaître J-F, Berger V, Bonenfant C, Devillard S, Douhard M, Gamelon M, Plard F, Lebreton J-D, Kliman RM (2016). Axes of variation in life histories. Encyclopedia of evolutionary biology.

[CR99] Gallach M, Betran E (2011). Intralocus sexual conflict resolved through gene duplication. Trends Ecol Evol.

[CR100] Garamszegi LZ, Mueller JC, Marko G, Szasz E, Zsebok S, Herczeg G, Eens M, Torok J (2014). The relationship between *DRD4* polymorphisms and phenotypic correlations of behaviors in the collared flycatcher. Ecol Evol.

[CR101] Garant D, Hadfield JD, Kruuk LEB, Sheldon BC (2008). Stability of genetic variance and covariance for reproductive characters in the face of climate change in a wild bird population. Mol Ecol.

[CR102] Gatewood JD, Wills A, Shetty S, Xy J, Arnold AP, Burgoyne PS, Rissman EF (2006). Sex chromosome complement and gonadal sex influence aggressive and parental behaviors in mice. J Neurosci.

[CR103] Gemmell NJ, Metcalf VJ, Allendorf FW (2004). Mother’s curse: the effect of mtDNA on individual fitness and population viability. Trends Ecol Evol.

[CR104] Gilbert LI, Schneiderman HA (1961). The content of juvenile hormone and lipid in Lepidoptera: sexual differences and developmental changes. Gen Comp Endocr.

[CR105] Glazier GS (2014). Is metabolic rate a universal ‘pacemaker’ for biological processes?. Biol Rev.

[CR106] Gosden TP, Chenoweth SF (2014). The evolutionary stability of cross-sex, cross-trait genetic covariances. Evolution.

[CR107] Gosden TP, Shastri KL, Innocenti P, Chenoweth SF (2012). The B-matrix harbors significant and sex-specific constraints on the evolution of multicharacter sexual dimorphism. Evolution.

[CR108] Gould SJ (1966). Allometry and size in ontogeny and phylogeny. Biol Rev.

[CR109] Grady DL, Harxhi A, Smith M, Flodman P, Spence MA, Swanson JM, Moyzis RK (2005). Sequence variants of the DRD4 gene in autism: further evidence that rare DRD4 7R haplotypes are ADHD specific. Am J Med Genet B Neuropsychiatr Genet..

[CR110] Grady DL, Thanos PK, Corrada MM (2013). *DRD4* genotype predicts longevity in mouse and human. J Neurosci.

[CR111] Grath S, Parsch J (2016). Sex-biased gene expression. Annu Rev Genet.

[CR112] Halloran J, Hussong SA, Burbank R (2012). Chronic inhibition of mammalian target of rapamycin by rapamycin modulates cognitive and non-cognitive components of behavior throughout lifespan in mice. Neuroscience.

[CR113] Hämäläinen A, Heistermann M, Kraus C (2015). The stress of growing old: sex- and season-specific effects of age on allostatic load in wild grey mouse lemurs. Oecologia.

[CR114] Hämäläinen A, Immonen E, Tarka M, Schuett W (2018) Evolution of sex-specific pace-of-life syndromes: causes and consequences. Behav Ecol Sociobiol: topical collection on Pace-of-life syndromes. 10.1007/s00265-018-2466-x 10.1007/s00265-018-2462-1 PMC5856903 29576676

[CR115] Han CS, Dingemanse NJ (2017). Sex-dependent expression of behavioral genetics architectures and the evolution of sexual dimorpshim. Proc R Soc Lon B.

[CR116] Handa RJ, Burgess LH, Kerr JE, Okeefe JA (1994). Gonadal-steroid hormone receptors and sex-differences in the hypothalamo-pituitary-adrenal axis. Horm Behav.

[CR117] Hansen TF, Houle D (2008). Measuring and comparing evolvability and constraint in multivariate characters. J Evol Biol.

[CR118] Harrison PW, Wright AE, Zimmer F, Dean R, Montgomery SH, Pointer MA, Mank JE (2015). Sexual selection drives evolution and rapid turnover of male gene expression. P Natl Acad Sci USA.

[CR119] Hartmann B, Castelo R, Minana B, Peden E, Blanchette M, Rio DC, Singh R, Valcarcel J (2011). Distinct regulatory programs establish widespread sex-specific alternative splicing in *Drosophila melanogaster*. RNA.

[CR120] Hau M (2007). Regulation of male traits by testosterone: implications for the evolution of vertebrate life histories. BioEssays.

[CR121] Hau M, Goymann W (2015). Endocrine mechanisms, behavioral phenotypes and plasticity: known relationships and open questions. Front Zool.

[CR122] Hayward AD (2013). Causes and consequences of intra- and inter-host heterogeneity in defence against nematodes. Parasite Immunol.

[CR123] Hollis B, Houle D, Yan Z, Kawecki TJ, Keller L (2014). Evolution under monogamy feminizes gene expression in *Drosophila melanogaster*. Nat Commun.

[CR124] Holmes A, Li Q, Murphy DL, Gold E, Crawley JN (2003). Abnormal anxiety-related behaviour in serotonin transporter null mutant mice: the influence of genetic background. Genes Brain Behav.

[CR125] Holtmann B, Grosser S, Lagisz M, Johnson SL, Santos ESA, Lara CE, Robertson BC, Nakagawa S (2016). Population differentiation and behavioural association of the two “personality” genes DRD4 and SERT in dunnocks (*Prunella modularis*). Mol Ecol.

[CR126] Horton BM, Hudson WH, Ortlund EA, Shirk S, Thomas JW, Young ER, Zinzow-Kramer WM, Maney DL (2014). Estrogen receptor alpha polymorphism in a species with alternative behavioral phenotypes. Proc Natl Acad Sci USA.

[CR127] Holtmann B, Grosser S, Lagisz M, Johnson SL, Santos ES, Lara CE, Robertson BC, Nakagawa S (2016). Population differentiation and behavioural association of the two 'personality' genes DRD4 and SERT in dunnocks (Prunella modularis). Mol Ecol..

[CR128] Houle D (1991) Genetic Covariance of fitness correlates: What genetic correlations are made of and why it matters. 45(3): 630-648. DOI: 10.1111/j.1558-5646.1991.tb04334.x 10.1111/j.1558-5646.1991.tb04334.x 28568816

[CR129] Husby A, Schielzeth H, Forstmeier W, Gustafsson L, Qvarnstrom A (2013). Sex chromosome linked genetic variance and the evolution of sexual dimorphism of quantitative traits. Evolution.

[CR130] Immonen E, Snook RR, Ritchie MG (2014). Mating system variation drives rapid evolution of the female transcriptome in *Drosophila pseudoobscura*. Ecol Evol.

[CR131] Immonen E, Collet M, Goenaga J, Arnqvist G (2016). Direct and indirect genetic effects of sex-specific mitonuclear epistasis on reproductive ageing. Heredity.

[CR132] Immonen E, Ronn J, Watson C, Berger D, Arnqvist G (2016). Complex mitonuclear interactions and metabolic costs of mating in male seed beetles. J Evol Biol.

[CR133] Immonen E, Sayadi A, Bayram H, Arnqvist G (2017). Mating changes sexually dimorphic gene expression in the seed beetle *Callosobruchus maculatus*. Genome Biol Evol.

[CR134] Imwalle DB, Gustafsson JA, Rissman EF (2005). Lack of functional estrogen receptor beta influences anxiety behavior and serotonin content in female mice. Physiol Behav.

[CR135] Innocenti P, Morrow EH, Dowling DK (2011). Experimental evidence supports a sex-specific selective sieve in mitochondrial genome evolution. Science..

[CR136] Isaac JL (2005). Potential causes and life-history consequences of sexual size dimorphism in mammals. Mammal Rev.

[CR137] Iwasa Y, Pomiankowski A (2001). The evolution of X-linked genomic imprinting. Genetics.

[CR138] Jazin E, Cahill L (2010). Sex differences in molecular neuroscience: from fruit flies to humans. Nat Rev Neurosci.

[CR139] Jones G (1995). Molecular mechanisms of action of juvenile hormone. Annu Rev Entomol.

[CR140] Juntti SA, Tollkuhn J, Wu MV, Fraser EJ, Soderborg T, Tan S, Honda S, Harada N, Shah NM (2010). The androgen receptor governs the execution, but not programming, of male sexual and territorial behaviors. Neuron.

[CR141] Ketterson ED, Nolan V, Sandell M (2005). Testosterone in females: mediator of adaptive traits, constraint on sexual dimorphism, or both?. Am Nat.

[CR142] Killen SS, Marras S, Metcalfe NB, McKenzie DJ, Domenici P (2013). Environmental stressors alter relationships between physiology and behaviour. Trends Ecol Evol.

[CR143] Kim SY, Fargallo JA, Vergara P, Martínez-Padilla J (2013). Multivariate heredity of melanin-based coloration, body mass and immunity. Heredity..

[CR144] Kingsolver JG, Wiernasz DC (1987). Dissecting correlated characters—adaptive aspects of phenotypic covariation in melanization pattern of *Pieris* butterflies. Evolution.

[CR145] Kittilsen S, Schjolden J, Beitnes-Johansen I, Shaw JC, Pottinger TG, Sorensen C, Braastad BO, Bakken M, Overli O (2009). Melanin-based skin spots reflect stress responsiveness in salmonid fish. Horm Behav.

[CR146] Klingenberg CP (2008). Morphological integration and developmental modularity. Annu Rev Ecol Evol S.

[CR147] Kolluru GR, Chappell MA, Zuk M (2004). Sex differences in metabolic rates in field crickets and their dipteran parasitoids. J Comp Physiol B.

[CR148] Konarzewski M, Książek A (2012). Determinants of intra-specific variation in basal metabolic rate. J Comp Physiol B.

[CR149] Koolhaas JM, de Boer SF, Coppens CM, Buwalda B (2010). Neuroendocrinology of coping styles: towards understanding the biology of individual variation. Front Neuroendocrinol.

[CR150] Korsten P, Mueller JC, Hermannstadter C (2010). Association between *DRD4* gene polymorphism and personality variation in great tits: a test across four wild populations. Mol Ecol.

[CR151] Krams IA, Niemelä PT, Trakimas G (2017). Metabolic rate associates with, but does not generate covariation between, behaviours in western stutter-trilling crickets, *Gryllus integer*. Proc R Soc B.

[CR152] Krasnov BR, Khokhlova IS, Burdelov SA, Fielden LJ (2004). Metabolic rate and jump performance in seven species of desert fleas. J Insect Physiol.

[CR153] Kudielka BM, Kirschbaum C (2005). Sex differences in HPA axis responses to stress: a review. Biol Psychol.

[CR154] Kudwa AE, Rissman EF (2003). Double oestrogen receptor alpha and beta knockout mice reveal differences in neural oestrogen-mediated progestin receptor induction and female sexual behaviour. J Neuroendocrinol.

[CR155] Kudwa AE, Michopoulos V, Gatewood JD, Rissman EF (2006). Roles of estrogen receptors alpha and beta in differentiation of mouse sexual behavior. Neuroscience.

[CR156] Kupper C, Stocks M, Risse JE (2016). A supergene determines highly divergent male reproductive morphs in the ruff. Nat Genet.

[CR157] Kurbalija Novicic Z, Immonen E, Jelic M, AnEthelkovic M, Stamenkovic-Radak M, Arnqvist G (2014). Within-population genetic effects of mtDNA on metabolic rate in *Drosophila subobscura*. J Evol Biol.

[CR158] Lande R (1980). Sexual dimorphism, sexual selection, and adaptation in polygenic characters. Evolution.

[CR159] Lande R, Arnold SJ (1983). The measurement of selection on correlated characters. Evolution.

[CR160] Lank DB, Smith CM, Hanotte O, Burke T, Cooke F (1995). Genetic polymorphism for alternative mating-behavior in lekking male ruff *Philomachus pugnax*. Nature.

[CR161] Lank DB, Coupe M, Wynne-Edwards KE (1999). Testosterone-induced male traits in female ruffs (*Philomachus pugnax*): autosomal inheritance and gender differentiation. Proc R Soc Lond B.

[CR162] Lank DB, Farrell LL, Burke T, Piersma T, McRae SB (2013). A dominant allele controls development into female mimic male and diminutive female ruffs. Biol Lett.

[CR163] Le Couteur DG, Solon-Biet S, Cogger VC, Mitchell SJ, Senior A, de Cabo R, Raubenheimer D, Simpson SJ (2016). The impact of low-protein high-carbohydrate diets on aging and lifespan. Cell Mol Life Sci.

[CR164] Lee KA (2006). Linking immune defenses and life history at the levels of the individual and the species. Integr Comp Biol.

[CR165] Lehtonen J, Parker GA, Scharer L (2016). Why anisogamy drives ancestral sex roles. Evolution.

[CR166] Lemos B, Araripe LO, Hartl DL (2008). Polymorphic Y chromosomes harbor cryptic variation with manifold functional consequences. Science.

[CR167] Lenormand T (2003). The evolution of sex dimorphism in recombination. Genetics.

[CR168] Lensing CJ, Adank DN, Doering SR, Wilber SL, Andreasen A, Schaub JW, Xiang Z, Haskell-Luevano C (2016). Ac-Trp-DPhe(p-I)-Arg-Trp-NH2, a 250-fold selective melanocortin-4rReceptor (MC4R) antagonist over the melanocortin-3 Receptor (MC3R), affects energy homeostasis in male and female mice differently. ACS Chem Neurosci..

[CR169] Lighthall NR, Mather M, Gorlick MA (2009). Acute stress increases sex differences in risk seeking in the balloon analogue risk task. PLoS One.

[CR170] Løvlie H, Immonen E, Gustavsson E, Kazancioglu E, Arnqvist G (2014) The influence of mitonuclear genetic variation on personality in seed beetles. Proc R Soc B 281:20141039. 10.1098/rspb.2014.1039 10.1098/rspb.2014.1039 PMC4213632 25320161

[CR171] Lund TD, Rovis T, Chung WCJ, Handa RJ (2005). Novel actions of estrogen receptor-beta on anxiety-related behaviors. Endocrinology.

[CR172] Luo JN, Lushchak OV, Goergen P, Williams MJ, Nassel DR (2014). *Drosophila* insulin-producing cells are differentially modulated by serotonin and octopamine receptors and affect social behavior. PLoS One.

[CR173] Lynch M, Walsh B (1998). Genetics and analysis of quantitative traits.

[CR174] Mafli A, Wakamatsu K, Roulin A (2011). Melanin-based coloration predicts aggressiveness and boldness in captive eastern Hermann’s tortoises. Anim Behav.

[CR175] Magurran AE, Garcia M (2000). Sex differences in behaviour as an indirect consequence of mating system. J Fish Biol.

[CR176] Magwere T, Chapman T, Partridge L (2004). Sex differences in the effect of dietary restriction on life span and mortality rates in female and male *Drosophila melanogaster*. J Gerontol A.

[CR177] Maklakov AA, Lummaa V (2013). Evolution of sex differences in lifespan and aging: causes and constraints. BioEssays.

[CR178] Maklakov AA, Simpson SJ, Zajitschek F, Hall MD, Dessmann J, Clissold F, Raubenheimer D, Bonduriansky R, Brooks RC (2008). Sex-specific fitness effects of nutrient intake on reproduction and lifespan. Curr Biol..

[CR179] Mank JE (2009). Sex chromosomes and the evolution of sexual dimorphism: lessons from the genome. Am Nat.

[CR180] Mank JE (2017). The transcriptional architecture of phenotypic dimorphism. Nat Ecol Evol.

[CR181] Mank JE, Axelsson E, Ellegren H (2007). Fast-X on the Z: rapid evolution of sex-linked genes in birds. Genome Res.

[CR182] Mank JE, Hosken DJ, Wedell N (2014). Conflict on the sex chromosomes: cause, effect, and complexity. Cold Spring Harb Perspect Biol.

[CR183] Masoro EJ (2005). Overview of caloric restriction and ageing. Mech Ageing Dev.

[CR184] Mateos-Gonzalez F, Senar JC (2012). Melanin-based trait predicts individual exploratory behaviour in siskins, *Carduelis spinus*. Anim Behav.

[CR185] Maures TJ, Booth LN, Benayoun BA, Izrayelit Y, Schroeder FC, Brunet A (2014). Males shorten the life span of *C. elegans* hermaphrodites via secreted compounds. Science.

[CR186] Maynard Smith J (1982). Evolution and the theory of games.

[CR187] McGlothlin JW, Jawor JM, Ketterson ED (2007). Natural variation in a testosterone-mediated trade-off between mating effort and parental effort. Am Nat.

[CR188] Melis MR, Succu S, Sanna F, Melis T, Mascia MS, Enguehard-Gueiffier C, Hubner H, Gmeiner P, Gueiffier A, Argiolas A (2006). PIP3EA and PD-168077, two selective dopamine D4 receptor agonists, induce penile erection in male rats: site and mechanism of action in the brain. Eur J Neurosci.

[CR189] Meunier J, Figueiredo Pinto S, Burri R, Roulin A (2010). Eumelanin-based coloration and fitness parameters in birds: a meta-analysis. Behav Ecol Sociobiol.

[CR190] Mills SC, Koskela E, Mappes T (2012). Intralocus sexual conflict for fitness: sexually antagonistic alleles for testosterone. Proc R Soc Lond B.

[CR191] Mokkonen M, Kokko H, Koskela E, Lehtonen J, Mappes T, Martiskainen H, Mills SC (2011). Negative frequency-dependent selection of sexually antagonistic alleles in *Myodes glareolus*. Science.

[CR192] Moshitzky P, Fleischmann I, Chaimov N, Saudan P, Klauser S, Kubli E, Applebaum SW (1996). Sex-peptide activates juvenile hormone biosynthesis in the *Drosophila melanogaster* corpus allatum. Arch Insect Biochem.

[CR193] Muehlenbein MP, Bribiescas RG (2005). Testosterone: mediated immune functions and male life histories. Am J Hum Biol.

[CR194] Mueller JC, Partecke J, Hatchwell BJ, Gaston KJ, Evans KL (2013). Candidate gene polymorphisms for behavioural adaptations during urbanization in blackbirds. Mol Ecol.

[CR195] Mueller JC, Edelaar P, Carrete M, Serrano D, Potti J, Blas J, Dingemanse NJ, Kempanaers B, Tella JL (2014). Behaviour-related *DRD4* polymorphisms in invasive bird populations. Mol Ecol.

[CR196] Mundy NI (2005). A window on the genetics of evolution: *MC1R* and plumage colouration in birds. Proc R Soc Lond B.

[CR197] Nijhout HF, Wheeler DE (1982). Juvenile hormone and the physiological basis of insect polymorphisms. Q Rev Biol.

[CR198] Ogawa S, Chester AE, Hewitt SC, Walker VR, Gustafsson JA, Smithies O, Korach KS, Pfaff DW (2000). Abolition of male sexual behaviors in mice lacking estrogen receptors alpha and beta (alpha beta ERKO). P Natl Acad Sci USA.

[CR199] Øverli Ø, Sørensen C, Nilsson GE (2006). Behavioral indicators of stress-coping style in rainbow trout: do males and females react differently to novelty?. Physiol Behav.

[CR200] Øverli Ø, Sørensen C, Pulman KGT, Pottinger TG, Korzan W, Summers CH, Nilsson GE (2007). Evolutionary backgrounds for stress-coping styles: relationshipps between physiological, behavioral and cognitive traits in non-mammalian vertebrates. Neurosci Biobehav Rev.

[CR201] Paaby AB, Rockman MV (2013). The many faces of pleiotropy. Trends Genet.

[CR202] Panaitof SC, Scott MP, Borst DW (2004). Plasticity in juvenile hormone in male burying beetles during breeding: physiological consequences of the loss of a mate. J Insect Physiol.

[CR203] Parker GA (1979). Sexual selection and sexual conflict.

[CR204] Perez-Barberia FJ, Perez-Fernandez E, Robertson E, Alvarez-Enriquez B (2008). Does the Jarman-Bell principle at intra-specific level explain sexual segregation in polygynous ungulates? Sex differences in forage digestibility in Soay sheep. Oecologia.

[CR205] Perry JC, Harrison PW, Mank JE (2014). The ontogeny and evolution of sex-biased gene expression in *Drosophila melanogaster*. Mol Biol Evol.

[CR206] Peters RH (1983). The ecological implications of body size.

[CR207] Poiani A (2006). Complexity of seminal fluid: a review. Behav Ecol Sociobiol.

[CR208] Pointer MA, Harrison PW, Wright AE, Mank JE (2013). Masculinization of gene expression is associated with exaggeration of male sexual dimorphism. PLoS Genet.

[CR209] Poissant J, Wilson AJ, Coltman DW (2009). Sex-specific genetic variance and the evolution of sexual dimorphism: a systematic review of cross-sex genetic correlations. Evolution.

[CR210] Raine A (2008). From genes to brain to antisocial behavior. Curr Dir Psychol Sci.

[CR211] Rantala MJ, Vainikka A, Kortet R (2003). The role of juvenile hormone in immune function and pheromone production trade-offs: a test of the immunocompetence handicap principle. Proc R Soc Lond B.

[CR212] Raskin K, de Gendt K, Duittoz A, Liere P, Verhoeven G, Tronche F, Mhaouty-Kodja S (2009). Conditional inactivation of androgen receptor gene in the nervous system: effects on male behavioral and neuroendocrine responses. J Neurosci.

[CR213] Réale D, Garant D, Humphries MM, Bergeron P, Careau V, Montiglio PO (2010). Personality and the emergence of the pace-of-life syndrome concept at the population level. Philos T Roy Soc B.

[CR214] Reeve JP, Fairbairn DJ (1996). Sexual size dimorphism as a correlated response to selection on body size: an empirical test of the quantitative genetic model. Evolution.

[CR215] Reinhold K, Engqvist L (2013). The variability is in the sex chromosomes. Evolution.

[CR216] Reinius B, Saetre P, Leonard JA, Blekhman R, Merino-Martinez R, Gilad Y, Jazin E (2008). An evolutionarily conserved sexual signature in the primate brain. PLoS Genet.

[CR217] Restif O, Amos W (2010). The evolution of sex-specific immune defences. Proc R Soc Lond B.

[CR218] Reynolds JD, Blackburn TM, Gaston KJ (2003). Life histories and extinction risk. Macroecology: concepts and consequences.

[CR219] Rice WR (1984). Sex-chromosomes and the evolution of sexual dimorphism. Evolution.

[CR220] Rice WR, Chippindale AK (2001). Intersexual ontogenetic conflict. J Evol Biol.

[CR221] Ricklefs RE, Wikelski M (2002). The physiology/life-history nexus. Trends Ecol Evol.

[CR222] Ritz KR, Noor MAF, Singh ND (2017). Variation in recombination rate: adaptive or not?. Trends Genet.

[CR223] Riyahi S, Sanchez-Delgado M, Calafell F, Monk D, Senar JC (2015) Combined epigenetic and intraspecific variation of the DRD4 and SERT genes influence novelty seeking behavior in great tit *Parus major*. Epigenetics 10:516–525. 10.1080/15592294.2015.1046027 10.1080/15592294.2015.1046027 PMC4622863 25933062

[CR224] Roberts ML, Buchanan KL, Evans MR (2004). Testing the immunocompetence handicap hypothesis: a review of the evidence. Anim Behav.

[CR225] Rogowitz GL, Chappell MA (2000). Energy metabolism of eucalyptus-boring beetles at rest and during locomotion: gender makes a difference. J Exp Biol.

[CR226] Rollins LA, Lee A, Whitehead MR, Woolnough AP, Sinclair R, Sherwin WB (2015). Is there evidence of selection in the *dopamine receptor D4* gene in Australian invasive starling populations?. Curr Zool.

[CR227] Rønning B, Moe B, Berntsen HH, Noreen E, Bech C (2014). Is the rate of metabolic ageing and survival determined by Basal metabolic rate in the zebra finch?. Plos One..

[CR228] Roulin A, Ducrest AL (2013). Genetics of colouration in birds. Semin Cell Dev Biol.

[CR229] Saccone G, Pane A, Polito LC (2002). Sex determination in flies, fruit flies and butterflies. Genetica.

[CR230] Saino N, Romano M, Rubolini D, Teplitsky C, Ambrosini R, Caprioli M, Canova L, Wakamatsu K (2013). Sexual dimorphism in melanin pigmentation, feather coloration and its heritability in the barnswallow (*Hirundo rustica*). PLoS One.

[CR231] Saltz JB, Hessel FC, Kelly MW (2017). Trait correlations in the genomics era. Trends Ecol Evol.

[CR232] Sapolsky RM (2005). The influence of social hierarchy on primate health. Science.

[CR233] Sapolsky RM, Romero LM, Munck AU (2000). How do glucocorticoids influence stress responses? Integrating permissive, suppressive, stimulatory, and preparative actions. Endocr Rev.

[CR234] Schal C, Holbrook GL, Bachmann JAS, Sevala VL (1997). Reproductive biology of the German cockroach, *Blattella germanica*: juvenile hormone as a pleiotropic master regulator. Arch Insect Biochem.

[CR235] Scheiner SM, Istock CA (1991). Correlational selection on life-history traits in the pitcher-plant mosquito. Genetica.

[CR236] Schinka JA, Letsch EA, Crawford FC (2002). DRD4 and novelty seeking: results of meta-analyses. Am J Med Genet..

[CR237] Schluter D (1996). Adaptive radiation along genetic lines of least resistance. Evolution.

[CR238] Schuett W, Tregenza T, Dall SR (2010). Sexual selection and animal personality. Biol Rev.

[CR239] Shelby JA, Madewell R, Moczek AP (2007). Juvenile hormone mediates sexual dimorphism in horned beetles. J Exp Zool B.

[CR240] Shih JC, Chen K (1999). MAO-A and -B gene knockout mice exhibit distinctly different behavior. Neurobiology.

[CR241] Shine R (1989). Alternative models for the evolution of offspring size. Am Nat.

[CR242] Sichova K, Koskela E, Mappes T, Lantova P, Boratynski Z (2014). On personality, energy metabolism and mtDNA introgression in bank voles. Anim Behav.

[CR243] Sih A, Bell AM, Johnson JC, Ziemba RE (2004). Behavioral syndromes: an integrative overview. Q Rev Biol.

[CR244] Silva JAP (1999). Sex hormones and glucocorticoids: interactions with the immune system. Ann N Y Acad Sci.

[CR245] Silva PIM, Martins CIM, Engrola S, Marino G, Overli O, Conceicao LEC (2010). Individual differences in cortisol levels and behaviour of Senegalese sole (*Solea senegalensis*) juveniles: evidence for coping styles. Appl Anim Behav Sci.

[CR246] Sinervo B, Svensson E (2002). Correlational selection and the evolution of genomic architecture. Heredity.

[CR247] Sinervo B, Svensson E, Comendant T (2000). Density cycles and an offspring quantity and quality game driven by natural selection. Nature.

[CR248] Singh P, Schimenti JC, Bolcun-Filas E (2015). A mouse geneticist’s practical guide to CRISPR applications. Genetics.

[CR249] Sirot LK, Wong A, Chapman T, Wolfner MF (2015). Sexual conflict and seminal fluid proteins: a dynamic landscape of sexual interactions. CSH Perspect Biol.

[CR250] Smith CA, Sinclair AH (2004). Sex determination: insights from the chicken. BioEssays.

[CR251] Sokal RR, Brussard PF (1978). Population differentiation: something new or more of the same?. Ecological genetics: the interface.

[CR252] Stamps JA (2007). Growth-mortality tradeoffs and ‘personality traits’ in animals. Ecol Lett.

[CR253] Stearns SC (1992). The evolution of life histories.

[CR254] Stillwell RC, Blanckenhorn WU, Teder T, Davidowitz G, Fox CW (2010). Sex differences in phenotypic plasticity affect variation in sexual size dimorphism in insects: from physiology to evolution. Annu Rev Entomol.

[CR255] Strambi A, Strambi C, Cayre M (1997). Hormonal control of reproduction and reproductive behavior in crickets. Arch Insect Biochem.

[CR256] Svensson E, Sinervo B, Comendant T (2001). Condition, genotype-by-environment interaction, and correlational selection in lizard life-history morphs. Evolution.

[CR257] Swanson EM, Dantzer B (2014). Insulin-like growth factor-1 is associated with life-history variation across Mammalia. Proc R Soc B.

[CR258] Tarka M, Akesson M, Hasselquist D, Hansson B (2014). Intralocus sexual conflict over wing length in a wild migratory bird. Am Nat.

[CR259] Tarka M, Guenther A, Niemelä PT, Nakagawa S, Noble DWA (2018) Sex differences in life-history, behavioral and physiological traits along a slow-fast continuum: a meta-analysis. Behav Ecol Sociobiol: topical collection on Pace-of-life syndromes. (in press) 10.1007/s00265-018-2534-2 PMC6060830 30100667

[CR260] Tatar M, Bartke A, Antebi A (2003). The endocrine regulation of aging by insulin-like signals. Science.

[CR261] Teplitsky C, Tarka M, Møller AP (2014). Assessing multivariate constraints to evolution across ten long-term avian studies. PLoS OnNE.

[CR262] Tower J (2006). Sex-specific regulation of aging and apoptosis. Mech Ageing Dev.

[CR263] Trumbo ST, Pfaff DW (2002). Hormonal regulation of parental care in insects. Hormones, brain, and behavior.

[CR264] Tudorache C, Schaaf MJM, Slabbekoorn H (2013). Covariation between behaviour and physiology indicators of coping style in zebrafish (*Danio rerio*). J Endocrinol.

[CR265] Tuttle EM, Bergland AO, Korody ML (2016). Divergence and functional degradation of a sex chromosome-like supergene. Curr Biol.

[CR266] van Dongen WFD, Robinson RW, Weston MA, Mulder RA, Guay PJ (2015). Variation at the *DRD4* locus is associated with wariness and local site selection in urban black swans. BMC Evol Biol.

[CR267] van Oers K, de Jong G, van Noordwijk AJ, Kempenaers B, Drent PJ (2005). Contribution of genetics to the study of animal personalities: a review of case studies. Behaviour.

[CR268] Veltsos P, Fang Y, Cossins AR, Snook RR, Ritchie MG (2017) Mating system manipulation and the evolution of sex-biased gene expression in Drosophila. Nat Commun 8:2072. 10.1038/s41467-017-02232-6. 10.1038/s41467-017-02232-6 PMC5727229 29233985

[CR269] Vieira C, Pasyukova EG, Zeng ZB, Hackett JB, Lyman RF, Mackay TF (2000). Genotype-environment interaction for quantitative trait loci affecting life span in *Drosophila melanogaster*. Genetics.

[CR270] Vinogradov AE (1998). Male reproductive strategy and decreased longevity. Acta Biotheor.

[CR271] Walsh B, Blows MW (2009). Abundant genetic variation plus strong selection = multivariate genetic constraints: a geometric view of adaptation. Annu Rev Ecol Evol S.

[CR272] Wedell N, Kvarnemo C, Tregenza T (2006). Sexual conflict and life histories. Anim Behav.

[CR273] Williams TD (2008). Individual variation in endocrine systems: moving beyond the ‘tyranny of the golden Mean’. Philos T Roy Soc B.

[CR274] Wolf M, van Doorn GS, Leimar O, Weissing FJ (2007). Life-history trade-offs favor the evolution of animal personalities. Nature.

[CR275] Wright AE, Darolti I, Bloch NI, Oostra V, Sandkam B, Buechel SD, Kolm N, Breden F, Vicoso B, Mank JE (2017). Convergent recombination suppression suggests role of sexual selection in guppy sex chromosome formation. Nat Commun.

[CR276] Wu MV, Shah NM (2011). Control of masculinization of the brain and behavior. Curr Opin Neurobiol.

[CR277] Wu MV, Manoli DS, Fraser EJ, Coats JK, Tollkuhn J, Honda SI, Harada N, Shah NM (2009). Estrogen masculinizes neural pathways and sex-specific behaviors. Cell.

[CR278] Wyatt GR, Davey KG (1996). Cellular and molecular actions of juvenile hormone. 2. Roles of juvenile hormone in adult insects. Adv Insect Physiol.

[CR279] Wyman MJ, Rowe L (2014). Male bias in distributions of additive genetic, residual, and phenotypic variances of shared traits. Am Nat.

[CR280] Wyman MJ, Wyman MC (2013). Sex-specific recombination rates and allele frequencies affect the invasion of sexually antagonistic variation on autosomes. J Evol Biol.

[CR281] Wyman MJ, Stinchcombe JR, Rowe L (2013). A multivariate view of the evolution of sexual dimorphism. J Evol Biol.

[CR282] Xu XH, Coats JK, Yang CF, Wang A, Ahmed OM, Alvarado M, Izumi T, Shah NM (2012). Modular genetic control of sexually dimorphic behaviors. Cell.

[CR283] Yamamoto R, Bai H, Dolezal AG, Amdam G, Tatar M (2013). Juvenile hormone regulation of *Drosophila* aging. BMC Biol.

[CR284] Zauner H, Begemann G, Mari-Beffa M, Meyer A (2003). Differential regulation of msx genes in the development of the gonopodium, an intromittent organ, and of the “sword,” a sexually selected trait of swordtail fishes (*Xiphophorus*). Evol Dev.

[CR285] Zuk M, Stoehr AM (2002). Immune defense and host life history. Am Nat.

[CR286] Zuloaga DG, Morris JA, Jordan CL, Breedlove SM (2008). Mice with the testicular feminization mutation demonstrate a role for androgen receptors in the regulation of anxiety-related behaviors and the hypothalamic-pituitary-adrenal axis. Horm Behav.

