# Supplementary material for: Evolution of sex-specific pace-of-life syndromes: genetic architecture and physiological mechanisms
Source: Behav Ecol Sociobiol. 2018 Mar 16;72(3):60. doi: 10.1007/s00265-018-2462-1 (PMC5856903; doi:10.1007/s00265-018-2462-1)
Supplement: Supplementary file 1 — (DOCX .5.5 kb) [file 265_2018_2462_MOESM1_ESM.docx]

**Behavioral Ecology and Sociobiology**

**Evolution of sex-specific pace-of-life syndromes: genetic architecture and physiological mechanisms**

Elina Immonen*^1^; Anni Hämäläinen^2^; Wiebke Schuett^3^; Maja Tarka^4^

**^1^** Department of Ecology and Genetics, Evolutionary Biology Centre (Animal Ecology Group), Uppsala University, Norbyvägens 18 D, 75236 Sweden

**^2^** Department of Biological Sciences, University of Alberta, Edmonton, T6G 2E9, Canada

**^3^** Zoological Institute, University of Hamburg, Martin-Luther-King Platz 3, 20146 Hamburg, Germany

**^4^** Center for Biodiversity Dynamics, Department of Biology, Norwegian University of Science and Technology (NTNU), Høgskoleringen 5, 7491, Trondheim, Norway

* Corresponding author: elina.immonen@ebc.uu.se

**Supplementary Material: Literature cited in the Tables 1-2**

Arnqvist G, Dowling DK, Eady P, Gay L, Tregenza T, Tuda M, Hosken DJ (2010) Genetic architecture of metabolic rate: environment specific epistasis between mitochondrial and nuclear genes in an insect. Evolution 64:3354-3363

Arnqvist GA, Stojkovic B, Liljestrand-Rönn J, Immonen E (2017) The pace-of-life: A sex-specific link between metabolic rate and life history in bean beetles. Func Ecol. 1-11. Doi: 10.1111/1365-2435.12927

Albers, HE, (2015) Species, sex and individual differences in the vasotocin/vasopressin system: relationship to neurochemical signaling in the social behavior neural network. Front Neuroendocrinol. 36:49-71. Doi: 10.1016/j.yfrne.2014.07.001

Ayllon F, Kjærner-Semb E, Furmanek T, Wennevik V, Solberg MF, Dahle G, et al. (2015) The *vgll3* locus controls age at maturity in wild and domesticated atlantic salmon (*Salmo salar* L.) males. PLoS Genet 11(11): e1005628. <https://doi.org/10.1371/journal.pgen.1005628>

Bale T, Epperson N (2015) Sex differences and stress across the lifespan. Nat Neurosci. 18(10): 1413–1420. Doi:10.1038/nn.4112.

# Barrett CA, Modi ME, Zhang BC, Walum H, Inoue K, Young LJ (2014) Neonatal melanocortin receptor agonist treatment reduces play fighting and promotes adult attachment in prairie voles in a sex-dependent manner. Neuropharmacology 85:357-366. Doi: <https://doi.org/10.1016/j.neuropharm.2014.05.041>

Barson NJ, Aykanat T, Hindar K, Baranski M, Bolstad GH, Fiske P, Jacq C, Jensen AJ, Johnston SE, Karlsson S, Kent M, Oen TM, Niemela E, Nome T, Naesje TF, Orell P, Romakkaniemi A, Saegrov H, Urdal K, Erkinaro J, Lien S, Primmer CR (2015) Sex-dependent dominance at a single locus maintains variation in age at maturity in salmon. Nature 528:405-+

Beitchman JH, Mik HM, Ehtesham S, Douglas L, Kennedy JL (2004) MAOA and persistent, pervasive childhood aggression. Mol. Psychiatry 9:546–547

Bendesky A, Kwon YM, Lassance JM, Lewarch CL, Yao SQ, Peterson BK, He MX, Dulac C, Hoekstra HE (2017) The genetic basis of parental care evolution in monogamous mice. Nature 544:434

Ben Zion IZ, Tessler R, Cohen L, Lerer E, Raz Y, Bachner-Melman R, Gritsenko I, Nemanov L, Zohar AH, Belmaker RH, Benjamin J, Ebstein RP (2006) Polymorphisms in the *dopamine D4 receptor gene (DRD4)* contribute to individual differences in human sexual behavior: desire, arousal and sexual function. Mol Psychiatr 11:782-786

Bielsky IF, Hu SB, Young LJ (2005) Sexual dimorphism in the vasopressin system: lack of an altered behavioral phenotype in female V1a receptor knockout mice. Behav Brain Res 164: 132–136

Camus MF, Clancy DJ, Dowling DK (2012) Mitochondria, maternal inheritance, and male aging. Curr Biol 22:1717-1721

Carpenter JP, Garcia JR, Lumm (2011) Dopamine receptor genes predict risk preferences, time preferences, and related economic choices. J Risk Uncertain. 42:233–261

Chan JSW, Snoeren EMS, Cuppen E, Waldinger MD, Olivier B, Oosting RS (2011) The Serotonin transporter plays an important role in male sexual behavior: a study in serotonin transporter knockout rats. J Sex Med 8:97-108

Chaki S, Okuyama S (2005) Involvement of melanocorti-4 receptor in anxiety and depression. Peptides 26(10): 1952-64

Cousminer DL et al (2013) Genome-wide association and longitudinal analyses reveal genetic loci linking pubertal height growth, pubertal timing and childhood adiposity. Hum Mol Genet 22(13):2735-47. doi: 10.1093/hmg/ddt104.

Coyne SP, Lindell SG, Clemente J, Barr CS, Parker KJ, Maestripieri D (2015) *Dopamine D4* receptor genotype variation in free-ranging rhesus macaques and its association with juvenile behavior. Behavioral brain research 292:50-55

Cushing BS, Perry A, Musatov S, Ogawa S, Papademetriou E (2008) Estrogen receptors in the medial amygdala inhibit the expression of male prosocial behavior. J Neurosci 28:10399-10403

# Dmitrieva J, Chen C, Greenberger E, Ogunseitan O, Ding Y (2011) Gender-specific expression of the *DRD4* gene on adolescent delinquency, anger and thrill seeking. Soc Cogn Affect Neurosci 6(1): 82–89. doi:  [10.1093/scan/nsq020](https://dx.doi.org/10.1093%2Fscan%2Fnsq020)

Ducrest AL, Keller L, Roulin A (2008) Pleiotropy in the melanocortin system, coloration and behavioural syndromes. Trends Ecol Evol 23:502-10

Dumais KM, Veenema AH (2016) Vasopressin and oxytocin receptor systems in the brain: sex differences and sex-specific regulation of social behavior. Front Neuroendocrinol. 40: 1-23

Dobler R, Rogell B, Budar F, Dowling DK (2014) A meta-analysis of the strength and nature of cytoplasmic genetic effects. J Evolution Biol 27:2021-34

Dordevic M, Stojkovic B, Savkovic U, Immonen E, Tucic N, Lazarevic J, Arnqvist G (2017) Sex-specific mitonuclear epistasis and the evolution of mitochondrial bioenergetics, ageing, and life history in seed beetles. Evolution 71:274-288

Emlen DJ, Warren IA, Johns A, Dworkin I, Lavine LC (2012) A mechanism of extreme growth and reliable signaling in sexually selected ornaments and weapons. Science 337:860-4

# Gangoso L, Grande JM, Ducrest A-L, Figuerola J, Bortolotti GR, Andres JA, Roulin A (2011) MC1R-dependent, melanin-based colour polymorphism is associated with cell-mediated response in the Eleonora’s falcon. JEB 24(1):2055-2063.

Gantz I, Fong TM (2003). The melanocortin system. Am J Phys - Endocrinology and Metabolism. 284(3): E468-E474 DOI:10.1152/ajpendo.00434.2002

Garamszegi LZ, Mueller JC, Marko G, Szasz E, Zsebok S, Herczeg G, Eens M, Torok J (2014) The relationship between *DRD4* polymorphisms and phenotypic correlations of behaviors in the collared flycatcher. Ecol Evol. 4:1466-1479

Goodin, S. Z., Keichler, A. R., Smith, M., Wendt, D., and Strader, A. D. (2008) Effect of gonadectomy on AgRP-induced weight gain in rats. Am. J. Physiol 295: R1747– R1753, DOI: 10.1152/ajpregu.90345.2008

Grady DL, Chi HC, Ding YC, Smith M, Wang E, Schuck S, Flodman P, Spence MA, Swanson JM, Moyzis RK (2003) High prevalence of rare *dopamine receptor D4* alleles in children diagnosed with attention-deficit hyperactivity disorder. Mol Psychiatr 8:536-545

Grady DL, Thanos PK, Corrada MM, Barnett JC, Ciobanu V, Shustarovich D, Napoli A, Moyzis AG, Grandy D, Rubinstein M, Wang GJ, Kawas CH, Chen CS, Dong Q, Wang E, Volkow ND, Moyzis RK (2013) *DRD4* genotype predicts longevity in mouse and human. J Neurosci 33:286-U693

Guzmán YF, Tronson NC, Jovasevic V, Sato K, Guedea AL, Mizukami H, Nishimori K, Radulovic J (2013) Fear-enhancing effects of septal oxytocin receptors. Nature Neuroscience. 16 (9): 1185–7. [doi](https://en.wikipedia.org/wiki/Digital_object_identifier):[10.1038/nn.3465](https://doi.org/10.1038%2Fnn.3465).

Faraone SV, Doyle AE, Mick E, Biederman J (2001) Meta-analysis of the association between the 7-repeat allele of the *dopamine D-4 receptor* gene and attention deficit hyperactivity disorder. Am J Psychiat 158:1052-1057

Feldman R, Bakermans-Kranenburg MJ. 2017. Oxytocin: a parenting hormone. Curr Opin Psychology 15:13-18. Doi: <https://doi.org/10.1016/j.copsyc.2017.02.011>

Fidler AE, van Oers K, Drent PJ, Kuhn S, Mueller JC, Kempenaers B (2007) *DRD4* gene polymorphisms are associated with personality variation in a passerine bird. Proc R Soc Lond B 274: 1685-1691.

Heijas K, Vas J, Topal J, Szantai E, Ronai Z, Szekely A, Kubinyi E, Horvath Z, Sasvari-Szekely M, Miklosi A (2007) Association of polymorphisms in the *Dopamine D4* receptor gene and the activity-impulsivity endophenotype in dogs. Anim Genet 38(6):629-33

Heinrichs M and Domes G (2008) Neuropeptides and social behaviour: effects of oxytocin and vasopressin in humans. Prog. Brain Research 170:337-350**.** Doi**:** <https://doi.org/10.1016/S0079-6123(08)00428-7>

# Holley A, Bellevue S, Vosberg D, Wenzel K, Roorda S Jr, Pfaus JG (2015) The role of oxytocin and vasopressin in conditioned mate guarding behavior in the female rat. Psyiol Behav 144:7-14. doi: 10.1016/j.physbeh.2015.02.039

Holtmann B, Grosser S, Lagisz M, Johnson SL, Santos ESA, Lara CE, Robertson BC, Nakagawa S (2016) Population differentiation and behavioural association of the two “personality” genes DRD4 and SERT in dunnocks (*Prunella modularis*). Molecular ecology 25:706-722

Horton BM, Hudson WH, Ortlund EA, Shirk S, Thomas JW, Young ER, Zinzow-Kramer WM, Maney DL (2014) Estrogen receptor alpha polymorphism in a species with alternative behavioral phenotypes. PNAS 111:1443-1448

Hull EM, Muschamp JW, Sato S (2004) Dopamine and serotonin: influences on male sexual behavior. Physiology & behavior 83:291-307

Immonen E, Collet M, Goenaga J, Arnqvist G (2016) Direct and indirect genetic effects of sex-specific mitonuclear epistasis on reproductive ageing. Heredity 116:338-47

Immonen E, Ronn J, Watson C, Berger D, Arnqvist G (2016) Complex mitonuclear interactions and metabolic costs of mating in male seed beetles. J Evolution Biol 29:360-370

Jannini EA, Burri A, Jern P, Novelli G (2015) Genetics of human sexual behavior: where we are, where we are going. Sex Med Rev 3:65-77

Kelly AM, Goodson JL (2013) Hypothalamic oxytocin and vasopressin neurons exert sex-specific effects on pair bonding, gregariousness, and aggression in finches. PNAS 111:6069-6074. Doi: 10.1073/pnas.1322554111

Kim SY, Fargallo JA, Vergara P, Martínez-Padilla J (2013) Multivariate heredity of melanin-based coloration, body mass and immunity. Heredity 111:139–146

Kim-Cohen J, Caspi A, Taylor A, Williams B, Newcombe R, Craig IW (2006). MAOA, maltreatment, and gene–environment interaction predicting children’s mental health: New evidence and a meta-analysis. Molecular Psychiatry 11: 903–913. doi:10.1038/sj.mp.4001851

Korsten P, Mueller JC, Hermannstadter C, Bouwman KM, Dingemanse NJ, Drent PJ, Liedvogel M, Matthysen E, van Oers K, van Overveld T, Patrick SC, Quinn JL, Sheldon BC, Tinbergen JM, Kempenaers B (2010) Association between *DRD4* gene polymorphism and personality variation in great tits: a test across four wild populations. Molecular ecology 19:832-843

Kriegsfeld LJ, Dawson TM, Dawson VL, Nelson RJ, Snyder SH (1997) Aggressive behavior in male mice lacking the gene for nNOS is testosterone-dependent. Brain Res 769:66–70

Kuo TH, Fedina TY, Hansen I *et al*. (2012) Insulin signaling mediates sexual attractiveness in *Drosophila*. PLoS Genet. 8: e1002684.

# Lensing CJ, Adank DN, Doering SR, Wilber SL, Andreasen A, Schaub JW, Xiang Z, Haskell-Luevano (2016) Ac-Trp-DPhe(p-I)-Arg-Trp-NH_2_, a 250-Fold Selective Melanocortin-4 Receptor (MC4R) antagonist over the Melanocortin-3 Receptor (MC3R), affects energy homeostasis in male and female mice differently. ACS Chem. Neurosci *7*(9): pp 1283–1291. DOI: 10.1021/acschemneuro.6b00156

Li DW, Sham PC, Owen MJ, He L (2006) Meta-analysis shows significant association between dopamine system genes and attention deficit hyperactivity disorder (ADHD). Hum Mol Genet 15:2276-2284

# Li K, Nakajima M, Ibanez-Tallon I, Heintz N (2016) A cortical circuit for sexually dimorphic oxytocin-dependent anxiety behaviors. Cell 167(1) 60-72. doi:  [10.1016/j.cell.2016.08.067](https://dx.doi.org/10.1016%2Fj.cell.2016.08.067)

Lovlie H, Immonen E, Gustavsson E, Kazancioglu E, Arnqvist G (2014) The influence of mitonuclear genetic variation on personality in seed beetles. Proc Biol Sci 281:20141039

Manuck SB, Flory JD, Ferrell RE, Mann JJ, Muldoon MF (2000) A regulatory polymorphism of the monoamine oxidase-A gene may be associated with variability in aggression, impulsivity, and central nervous system responsivity. Psychiatry Res. 95:9–23

McDermot R, Tingley D, Cowden J, Frazzetto G, Johnson DDP (2009). Monoamine oxidase A gene (MAOA) predicts behavioral aggression following provocation. PNAS 106: 2118-2123.

Melis MR, Succu S, Sanna F, Melis T, Mascia MS, Enguehard-Gueiffier C, Hubner H, Gmeiner P, Gueiffier A, Argiolas A (2006) PIP3EA and PD-168077, two selective dopamine D4 receptor agonists, induce penile erection in male rats: site and mechanism of action in the brain. Eur J Neurosci 24:2021-2030

Mueller JC, Partecke J, Hatchwell BJ, Gaston KJ, Evans KL (2013) Candidate gene polymorphisms for behavioural adaptations during urbanization in blackbirds. Mol Ecol 22:3629-3637

Mueller JC, Edelaar P, Carrete M, Serrano D, Potti J, Blas J, Dingemanse NJ, Kempanaers B, Tella JL (2014) Behaviour-related *DRD4* polymorphisms in invasive bird populations. Mol Ecol 23:2876-2885

[Munafò MR](https://www.ncbi.nlm.nih.gov/pubmed/?term=Munaf%C3%B2%20MR%5BAuthor%5D&cauthor=true&cauthor_uid=17574217), [Yalcin B](https://www.ncbi.nlm.nih.gov/pubmed/?term=Yalcin%20B%5BAuthor%5D&cauthor=true&cauthor_uid=17574217), [Willis-Owen SA](https://www.ncbi.nlm.nih.gov/pubmed/?term=Willis-Owen%20SA%5BAuthor%5D&cauthor=true&cauthor_uid=17574217), [Flint J](https://www.ncbi.nlm.nih.gov/pubmed/?term=Flint%20J%5BAuthor%5D&cauthor=true&cauthor_uid=17574217). 2008 Association of the Dopamine D4 Receptor (DRD4) gene and approach-related personality traits: meta-analysis and new data. Biol Psychiatry 63(2): 197-206

Nelson RJ Demas GE, Huang PL, Fishman MC, Dawson VL, Dawson TM, Snyder SH (1995) Behavioural abnormalities in male mice lacking neuronal nitric oxide synthase. Nature 378, 383–386.

Nelson RJ & Trainor BC (2007). Neural mechanisms of aggression. Nature Reviews Neuroscience, 8, 536–546. doi:10.1038/nrn2174

Olivier B, Chan JSW, Snoeren EM, Olivier JDA, Veening JG, Vinkers CH, Waldinger MD, Oosting RS (2010) Differences in sexual behaviour in male and female rodents: Role of Serotonin . *In* Neill JC and Kulkarni J (eds.), Biological Basis of Sex Differences in Psychopharmacology. Curr Topics Behav Neur Sci 8, pp 15-36. DOI 10.1007/7854_2010_116

Parsey RV, Oquendo MA, Simpson NR, Ogden RD, Van Heertum R, Arango V, Mann JJ. (2002) Effects of sex, age, and aggressive traits in man on brain serotonin 5-HT_1A_ receptor binding potential measured by PET using [C-11]WAY-100635. Brain Research 954(2):173-182

Riyahi S, Sanchez-Delgado M, Calafell F, Monk D, Senar JC (2015) Combined epigenetic and intraspecific variation of the DRD4 and SERT genes influence novelty seeking behavior in great tit *Parus major*. Epigenetics 10:516-525

Schilling C, Kühn S, Sander T, Gallinat J (2014) Association between dopamine D4 receptor genotype and trait impulsiveness. Psychiatr Genet 24(2):82

Schinka JA, Letsch EA, Crawford FC (2002) *DRD4* and novelty seeking: results of meta-analyses. M J Med Genet 114(6):643-8

Scordalakes EM, Rissman EF (2003) Aggression in male mice lacking functional estrogen receptor alpha. Behav Neurosci.117(1):38-45.

Serretti A, Calati R, Mandelli L, De Ronchi D (2006) Serotonin transporter gene variants and behavior: a comprehensive review. Curr Drug Targets 7(12):1659-69

Shih JC, Chen K, Ridd MJ (1999) Monoamine oxidase: from genes to behavior. Annu. Rev. Neurosci. 22: 197–217

Sichova K, Koskela E, Mappes T, Lantova P, Boratynski Z (2014) On personality, energy metabolism and mtDNA introgression in bank voles. Anim Behav 92:229-237

Steinman MQ, Duque-Wilckens N, Greenberg GD, Hao R, Campi KL, Laredo SA, Laman-Maharg A, Manning CE, Doig IE, Lopez EM, Walch K, Bales KL, Trainor BC (2016) Biol Psychiatry 80(5): 406-14. Doi: 10.1016/j.biopsych.2015.10.007

Swanson EM, Dantzer B (2014) Insulin-like growth factor-1 is associated with life-history variation across Mammalia. Proc Roy Soc B-Biol Sci 281

##### Timm Tilgar C, Saag P (2015) DRD4 gene polymorphism in great tits: gender-specific association with behavioural variation in the wild. Behav Ecol Sociobiol 69:729–735

Trainor BC, Workman JL, Jessen R, Nelson RJ (2007) Impaired nitric oxide synthase signaling dissociates social investigation and aggression. Behav Neurosci 121: 362–369

Trainor BC, Lin S, Finy MS, Rowland MR, Nelson RJ (2007) Photoperiod reverses the effects of estrogen on male aggression via genomic and non-genomic pathways. PNAS*.* doi: 10.1073/pnas.0701819104

Toufexis DJ, Myers KM, Bowser ME, Davis M (2007) Estrogen disrupts the inhibition of fear in female rats, possibly through the antagonistic effects of estrogen receptor alpha (ERalpha) and ERbeta. J Neurosci 27(36):9729-35.

Van Dongen WFD, Robinson RW, Weston MA, Mulder RA, Guay PJ (2015) Variation at the *DRD4* locus is associated with wariness and local site selection in urban black swans. BMC Evol Biol 15: 253. doi:  [10.1186/s12862-015-0533-8](https://dx.doi.org/10.1186%2Fs12862-015-0533-8)

Verona E, Joiner TE, Johnson F, Bender TW (2006) Gender specific gene–environment interactions on laboratory-assessed aggression. Biol Psychol 71: 33–41
