# Supplementary material for: Evolution of sex-specific pace-of-life syndromes: genetic architecture and physiological mechanisms
Source: Behav Ecol Sociobiol. 2018 Mar 16;72(3):60. doi: 10.1007/s00265-018-2462-1 (PMC5856903; doi:10.1007/s00265-018-2462-1)
Supplement: Supplementary file 2 — (DOCX 476 kb) [file 265_2018_2462_MOESM2_ESM.docx]

**Behavioral Ecology and Sociobiology**

**Evolution of sex-specific pace-of-life syndromes: genetic architecture and physiological mechanisms**

Elina Immonen*^1^; Anni Hämäläinen^2^; Wiebke Schuett^3^; Maja Tarka^4^

**^1^** Department of Ecology and Genetics, Evolutionary Biology Centre (Animal Ecology Group), Uppsala University, Norbyvägens 18 D, 75236 Sweden

**^2^** Department of Biological Sciences, University of Alberta, Edmonton, T6G 2E9, Canada

**^3^** Zoological Institute, University of Hamburg, Martin-Luther-King Platz 3, 20146 Hamburg, Germany

**^4^** Center for Biodiversity Dynamics, Department of Biology, Norwegian University of Science and Technology (NTNU), Høgskoleringen 5, 7491, Trondheim, Norway

* Corresponding author: elina.immonen@ebc.uu.se

**Supplementary Figure 1**

**Fig S1** The POLS framework predicts covariation among life history, physiology and behavioral traits along the fast-slow continuum of pace-of-life. The sexes can differ in the mean expression of POLS traits along this continuum in such a way that the trait covariance across the sexes (i.e. B-matrix) forms the pace-of-life syndrome at the population level (black dotted line). In addition to sexual dimorphism in the trait means, the sexes may also differ in the within-sex trait covariances (i.e. G-matrix), which may or may not align with the trait covariance across sexes (solid and dashed lines, respectively). For illustrative purposes we here show an example of lack of covariance between traits A and B in one sex (orange dashed line) and a negative covariance in the other (green dashed line).​
